# Genetically Encoded Photocatalysis Enables Spatially Restricted Optochemical Modulation of Neurons in Live Mice

**DOI:** 10.1021/acscentsci.3c01351

**Published:** 2023-12-18

**Authors:** Kaixing Zeng, Zhi-Han Jiao, Qin Jiang, Ru He, Yixin Zhang, Wei-Guang Li, Tian-Le Xu, Yiyun Chen

**Affiliations:** † State Key Laboratory of Chemical Biology, Shanghai Institute of Organic Chemistry, University of Chinese Academy of Sciences, Chinese Academy of Sciences, 345 Lingling Road, Shanghai 200032 China; ‡ School of Physical Science and Technology, ShanghaiTech University, 100 Haike Road, Shanghai 201210, China; § Centre for Brain Science and Department of Anatomy and Physiology, Shanghai Jiao Tong University School of Medicine, 280 South Chongqing Road, Shanghai 200025, China; ∥ Department of Rehabilitation Medicine, Huashan Hospital, Institute for Translational Brain Research, State Key Laboratory of Medical Neurobiology and Ministry of Education Frontiers Centre for Brain Science, Fudan University, 131 Dongan Road, Shanghai 200032, China; ⊥ School of Chemistry and Material Sciences, Hangzhou Institute for Advanced Study, University of Chinese Academy of Sciences, Sub-lane Xiangshan, Hangzhou 310024, China

## Abstract

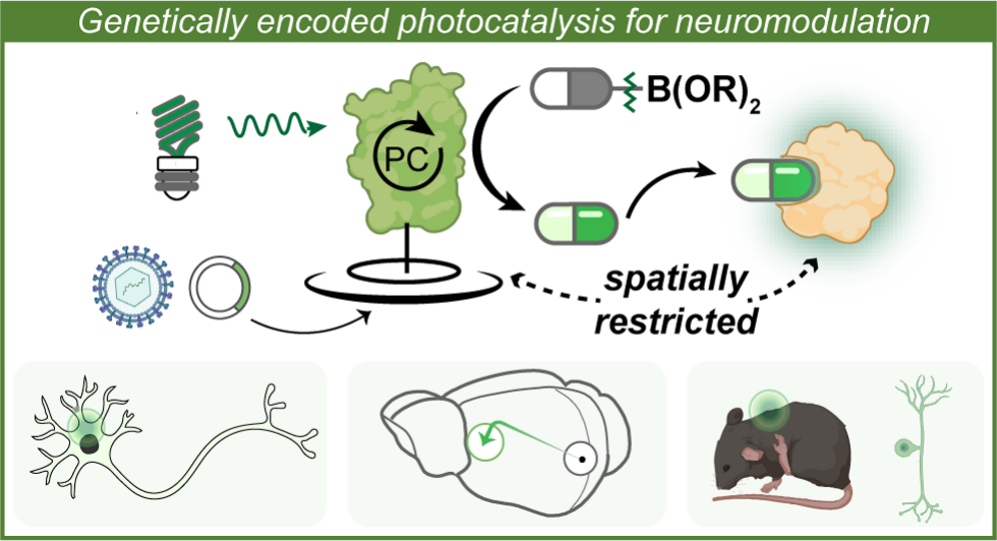

Light provides high temporal precision for neuronal modulations. Small molecules are advantageous for neuronal modulation due to their structural diversity, allowing them to suit versatile targets. However, current optochemical methods release uncaged small molecules with uniform concentrations in the irradiation area, which lack spatial specificity as counterpart optogenetic methods from genetic encoding for photosensitive proteins. Photocatalysis provides spatial specificity by generating reactive species in the proximity of photocatalysts. However, current photocatalytic methods use antibody-tagged heavy-metal photocatalysts for spatial specificity, which are unsuitable for neuronal applications. Here, we report a genetically encoded metal-free photocatalysis method for the optochemical modulation of neurons via deboronative hydroxylation. The genetically encoded photocatalysts generate doxorubicin, a mitochondrial uncoupler, and baclofen by uncaging stable organoboronate precursors. The mitochondria, nucleus, membrane, cytosol, and ER-targeted drug delivery are achieved by this method. The distinct signaling pathway dissection in a single projection is enabled by the dual optogenetic and optochemical control of synaptic transmission. The itching signaling pathway is investigated by photocatalytic uncaging under live-mice skin for the first time by visible light irradiation. The cell-type-specific release of baclofen reveals the GABA_B_R activation on Na_V_1.8-expressing nociceptor terminals instead of pan peripheral sensory neurons for itch alleviation in live mice.

## Abstract 2

We report the first genetically encoded photocatalysis method for spatially restricted optochemical modulation of neurons in live mice, with subcellular, projection, and cell-type specificity.

## Introduction

**Figure 1:**
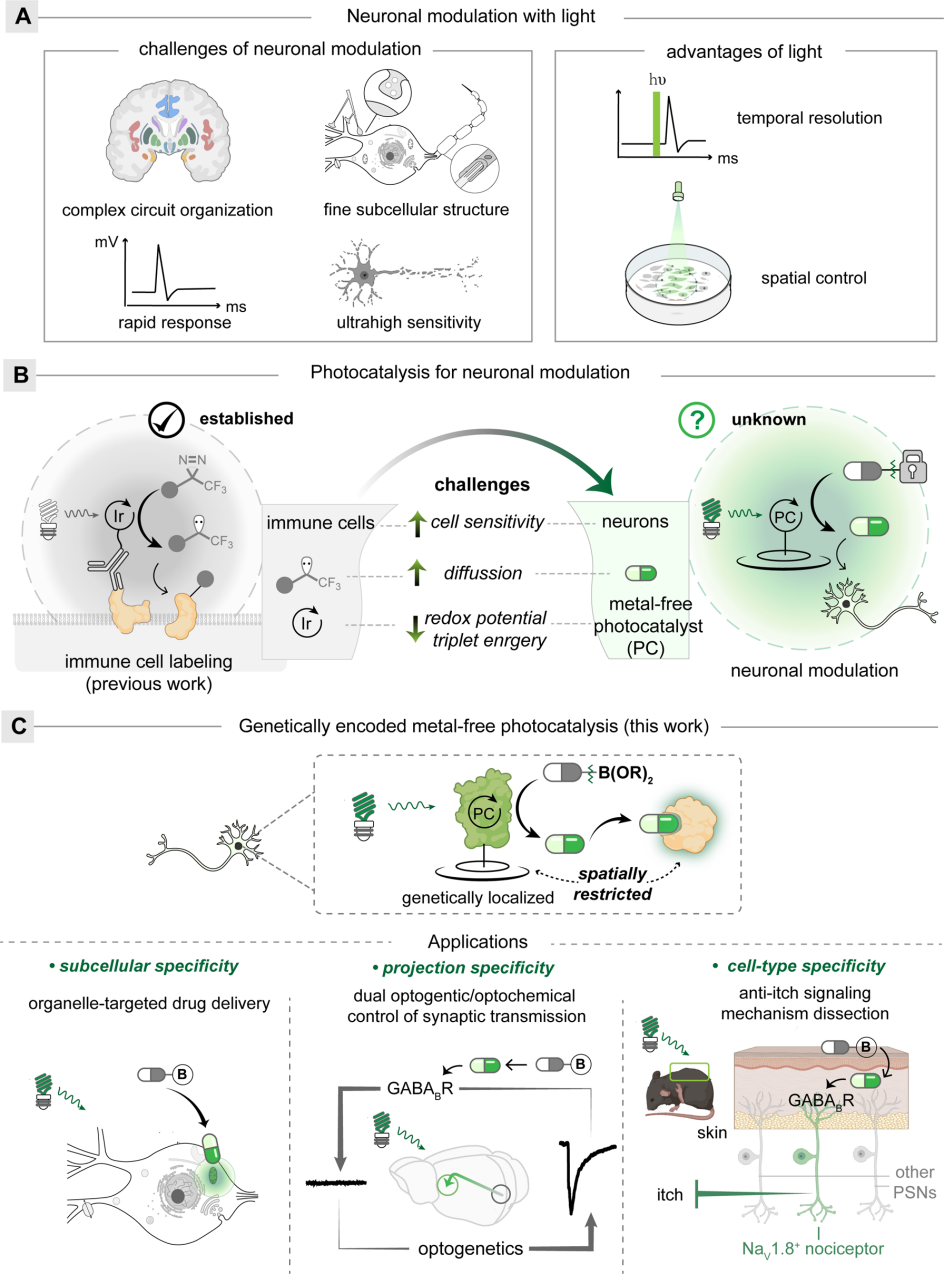
Neuronal modulation by photocatalysis. (A) Light provides spatiotemporal precision for neuronal modulation. (B) Challenges of neuronal modulation by photocatalysis. Heavy-metal photocatalysts for immune cell labeling with carbenes (established) vs metal-free photocatalysts for neuronal modulation with drug molecules (unknown). (C) Genetically encoded metal-free photocatalysis modulates neurons with spatial specificity.

Neurons have complex circuit organization, fine subcellular structures, and rapid response to environments, which pose significant challenges for on-demand *in vivo* modulations (Figure 1A).^1,2^ Light provides high temporal resolution for neuronal modulation.^3^ Among neuronal photomodulation techniques, optogenetics is revolutionary in utilizing photosensitive proteins to provide prompt neuronal responses to photon signals.^4,5^ It interrogates neuronal functions with subcellular, projection, and cell-type-specific photosensitive proteins by well-established genetic encoding tools. Despite the great success of optogenetic methods, there is a high demand for photomodulation techniques with small molecules (Figure 1B).^6,7^ The optochemical methods offer diverse structural properties by photouncaging small molecules to elucidate versatile biological functions.^8,9^ However, current optochemical methods use direct excitation on small molecules for photolytic uncaging.^10,11^ As a result, the uncaged small molecules have uniform concentrations in the illumination area, which is unsuitable for neuroscience research requiring subcellular, projection, and cell-type preference.^12,13^

In contrast, the photocatalytic methods only activate small molecules that are spatially proximal to the excited photocatalysts (Figure 1B).^14,15^ With the μMap method for proximal protein labeling, antibody-tagged iridium photocatalysts provide selective cell-membrane microenvironment mapping for cancer and immune cells.^16−18^ These photocatalytic reactions generate concentration gradients of reactive species radiating from the photocatalyst to provide spatial specificity. While effective, those methods were unsuitable for neuronal modulations due to the neuronal toxicity from heavy-metal photocatalysts and the poor neuronal specificity from antibody targeting.^19,20^ To achieve neuronal modulation with photocatalysis, key bottlenecks need to be conquered: *i*) The photocatalysis needs to be compatible with neurons, especially the compatibility of the ultrasensitive neurons to the unavoidable reactive oxygen species generation by photosensitization; *ii*) the photocatalytically generated uncaged small molecules need to provide spatial specificity as desired compared to photolytic uncaging; and *iii*) the metal-free photocatalysts in neurons need to uncage small molecules with efficiency comparable to that of heavy-metal photocatalysts. Herein, we report the first genetically encoded metal-free photocatalysis method to modulate neurons with small molecules after overcoming the above challenges (Figure 1C).

## Results

### Development of Genetically Encodable Photocatalysts with SNAP-tag and Organic Dyes

**Figure 2:**
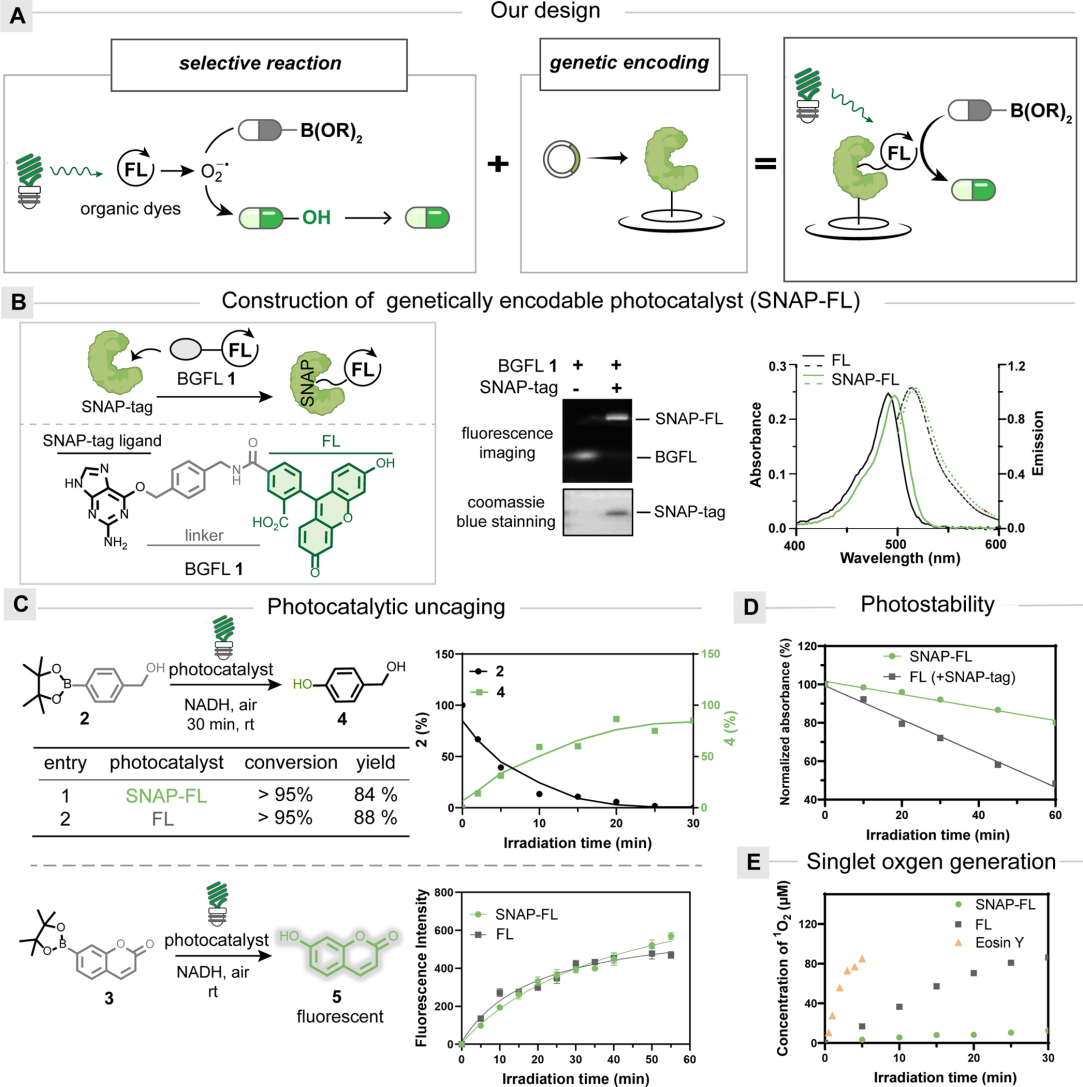
Development of genetically encoded photocatalysts with SNAP-tag and organic dyes. (A) Genetically encoded SNAP-FL protein uncages photoinsensitive organoboronates by selective deboronative hydroxylation. (B) SNAP-FL protein is constructed from SNAP-tag and BGFL. (Left) Chemical structure of BGFL. (Middle) Gel analysis of SNAP-FL protein. (Right) Absorption (solid line) and emission (dashed line) spectrum of SNAP-FL protein and fluorescein. (C) Kinetic studies of the photocatalytic uncaging of organoboronates **2** (top) and **3** (bottom) by SNAP-FL protein and fluorescein. Reaction conditions: 500 nm light irradiation (2.9 mW/cm^2^) in pH 7.4 PBS buffer for 30 min. (D) Photostability of SNAP-FL protein under 500 nm light irradiation (4.5 mW/cm^2^) compared to that of fluorescein. (E) Singlet oxygen generation by SNAP-FL protein is minimal compared to that of fluorescein and Eosin Y under 500 nm light irradiation (4.5 mW/cm^2^).

Organoboronic acids are stable and environmentally friendly, for which structural motifs are widely present in many clinically approved drugs.^21,22^ In addition, they are widely used for cellular hydrogen peroxide detection as molecular probes and diagnostic moieties.^23−26^ We previously identified organic dyes as selective photocatalysts in live cells while maintaining biocompatibility.^27,28^ These organic dyes, while having a lower redox capacity compared to that of iridium photocatalysts, can generate low concentrations of superoxide radical anions in a biocompatible and controllable manner upon visible light irradiation,^29,30^ which uncages small molecules by selective deboronative hydroxylation. We envision that linking photocatalytically active organic dyes with proteins of interest may construct the genetically encodable photocatalysts.^31^ The implementation of this concept will enable the optochemical modulation of neurons with genetically encoded spatial specificity, which is highly desirable but uncharted territory in the photomodulation of neurons (Figure 2A).

Our investigation began with synthesizing the small molecule probe BGFL **1**, containing O^6^-benzylguanine (BG) as the directing ligand and organic dye fluorescein (FL) for photocatalysis.^27,28^ After mixing equal concentrations of BGFL with the commercially available SNAP-tag,^32^ we observed the formation of the conjugated SNAP-FL protein quantitatively in 60 s (Figure 2B). The UV absorption and fluorescence emission spectra of the SNAP-FL protein closely resembled those of FL. To assess the photocatalytic capability of the SNAP-FL protein, we employed HPLC analysis to measure the conversion of *p*-boronic ester benzyl alcohol **2** by deboronative hydroxylation. In addition, the fluorescence emission of coumarin boronic ester **3** was measured to monitor its conversion. Under green light irradiation for 30 min, SNAP-FL protein exhibited excellent deboronative hydroxylation activity for **2** and **3**, achieving similar photocatalytic yields compared to that of the free FL (Figure 2C, Figure S3A, and Table S1). The crucial roles of light irradiation and photocatalyst were confirmed by the control experiments.

We next explored the photostability of SNAP-FL protein and found that more than 80% of SNAP-FL protein remained intact after 1 h of irradiation, indicating a significant improvement in the photostability comparable to that of free small-molecule FL (Figure 2D)^33−35^ Moreover, we measured singlet oxygen generation using 9,10-anthracenedipropanoic acid and observed that SNAP-FL protein generated very little singlet oxygen, which suggested excellent biocompatibility, especially for neuronal applications (Figure 2E).^36^ The fluorescence quenching experiments showed that the excited states of SNAP-FL protein and small-molecule fluorescein were quenched at similar rates by reductants, followed by the generation of superoxide radical anions to induce deboronative hydroxylation (Figure S2).^37^ We also quantified the photocatalytic generation of superoxide radical anions by SNAP-FL protein and found that the photocatalytically generated superoxide radical anions (∼10–100 μM) were far beyond their physiological levels in cells (8 pM, Figure S2).^38^ We also compared the reaction kinetics between the photocatalytic uncaging of organoboronic acids and hydrogen peroxide-induced uncaging, in which the former was much more effective (Figure S2).^39,40^

### Intracellular Protein Modulation with Organelle-Specific Doxorubicin Release

**Figure 3:**
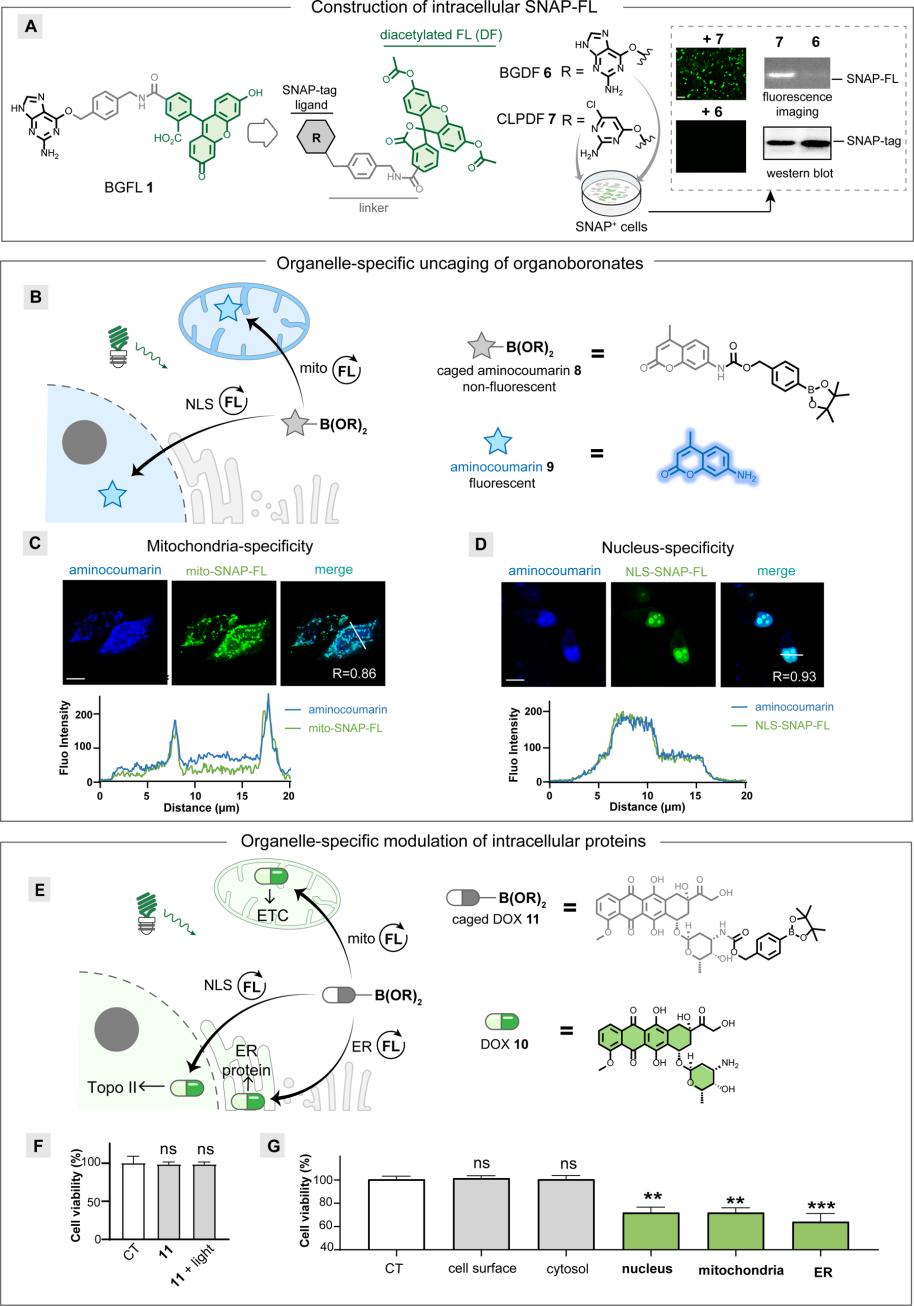
Intracellular protein modulation with organelle-specific drug release. (A) Switch of small-molecule BGFL to CLPDF for intracellular SNAP-FL protein construction. The intracellular SNAP-FL protein was characterized by imaging and electrophoresis (right). (B) Schematic representation of aminocoumarin **9** release from organoboronates **8** by selective photocatalysis in the nucleus and mitochondria. (C) mito-SNAP-FL protein releases aminocoumarin **9** from organoboronates **8** with mitochondria specificity. The colocalization between **9** and mito-SNAP-FL protein shows *R* = 0.86. (D) NLS-SNAP-FL protein releases aminocoumarin **9** from organoboronates **8** with nucleus specificity. The colocalization between **9** and NLS-SNAP-FL protein shows *R* = 0.93. Overlays of line profiles at the bottom in (C) and (D) show the pixel intensities along the indicated thin white lines. Scale bar: 10 μm. (E) Schematic representation of DOX **10** release from organoboronate caged-DOX **11** for the Topo II inhibition in the nucleus and electron transport chain (ETC) modulation in mitochondria. (F) Cell viability of organoboronate caged-DOX **11**. (G) Organelle-specific release of DOX **10** from organoboronate caged-DOX **11** in different subcellular compartments (*n* = 7). CT: control group. The statistical significance of differences between groups was evaluated with the unpaired Student’s *t* test. ns is not statistically significant. All *p* values were calculated with control cells treated without transfection, light, or small molecules. Data are shown as the mean ± S. E. M.

After successfully establishing the SNAP-FL protein for the photocatalytic uncaging of organoboronates in a cell-free environment, we next attempted the intracellular applications. Due to the negatively charged nature of FL, the small-molecule BGFL exhibits poor cell membrane penetration. We speculate that diacetylated fluorescein has improved cell membrane permeability, which upon cell penetration releases free fluorescein by the endogenous esterase hydrolysis.^41^ The diacetylated fluorescein-linked BGDF **6** was first synthesized, but the limited intracellular SNAP-FL protein formation was observed due to the low lipid solubility of BG (log P = 2.0). The diacetylated fluorescein-linked CLPDF **7** was next tested, bearing O4-benzyl-2-chloro-6-aminopyrimidine with improved lipid solubility (CLP, log P = 2.7).^42^ The live-cell imaging and gel analysis indicated that CLPDF **7** was more effective in constructing SNAP-FL proteins inside HeLa cells than BGDF **6** (Figure 3A).

We then transfected the mitochondria-localized mito-SNAP gene in HeLa cells and incubated with CLPDF **7**. Fluorescence imaging revealed the excellent colocalization of mito-SNAP-FL protein with the mitochondria marker TMRE (Figure S4). The boronate-caged aminocoumarin **8** was next incubated with HeLa cells expressing mito-SNAP-FL protein, and a clear colocalization of mito-SNAP-FL protein with aminocoumarin **9** was observed after 20 min of green light irradiation (Figure 3B). These colocalization results are attributed to the deboronative hydroxylation and 1,6-elimination of **8** catalyzed by mito-SNAP-FL proteins localized in the mitochondria (Figure 3C, Figure S5A). Similarly, nucleus-localized NLS-SNAP-FL proteins were constructed and tested with boronate-caged aminocoumarin **8** for nucleus-localized release. The colocalization of uncaged aminocoumarin **9** with the NLS-SNAP-FL protein clearly demonstrated the diversified subcellular specificity of this method (Figure 3D, Figure S5B).

Next, we used this method to investigate the subcellular targets of the anticancer drug doxorubicin (DOX) **10**. DOX is known to induce apoptosis and inhibit cell proliferation, which established biological targets including nuclear topoisomerase II (Topo II) and the mitochondrial electron transport chain (Figure 3E).^43,44^ We synthesized boronate-caged DOX **11** from DOX **10** in one step, which remains stable and shows no phototoxicity in the absence of a photocatalyst (Figure 3F).

The membrane-bound and cytosol-localized SNAP-FL proteins were first constructed in HeLa cells. Afterward, the uncaging of DOX **10** was performed in those cells and showed no significant biological effects.^45^ In contrast, for cells expressing the NLS-SNAP-FL protein or mito-SNAP-FL protein, the identical concentrations of boronate-caged DOX **11** resulted in significant cytotoxic effects (Figure 3G, Figure S6A,B). When the concentration of caged DOX **11** is increased or the light-irradiation time is extended for the cytosol-localized SNAP-FL proteins, decreased cell viability is observed. However, the cell viablity is still higher than that from the nucleus-released and mitochondria-released DOX (Figure S7).^46^

Interestingly, cells expressing endoplasmic reticulum (ER)-targeted ER-SNAP-FL protein also demonstrated significant cytotoxicity by DOX **10** release. While there are only isolated reports on the cardiotoxicity from DOX-induced ER stress, we expect that other ER-specific pathways will be disclosed by the biological perturbation with DOX (Figure 3G, Figure S6C).^47^ Together, the high spatial resolution demonstrated by the photocatalytic uncaging of small molecules echoes the click-to-release strategy for spatial specificity, suggesting further organelle-specific investigations with potential therapeutic interventions.^48,49^

### Neuronal Activity Modulation with Organelle-Specific 2,4-Dinitrophenol Release

**Figure 4:**
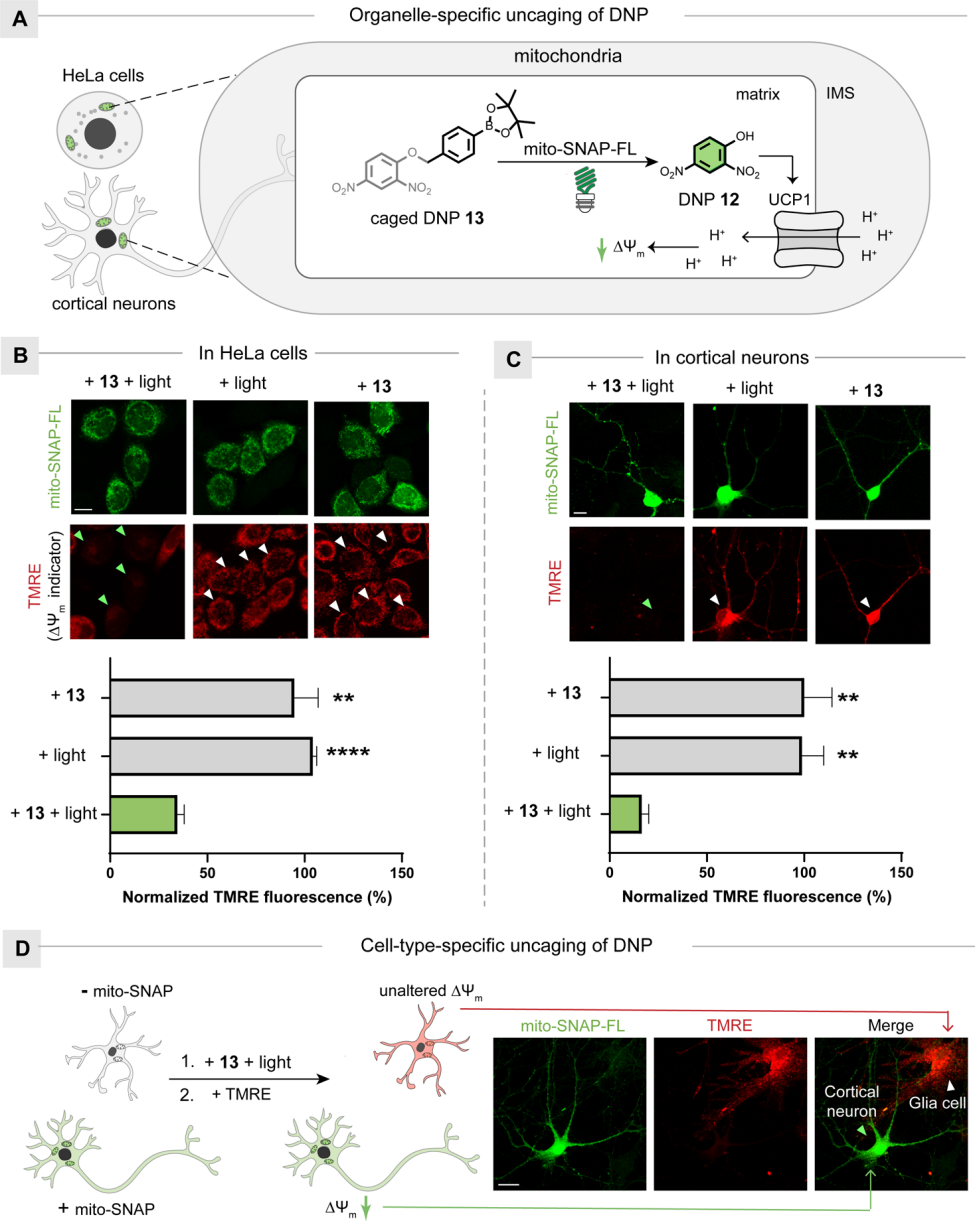
Neuronal activity modulation with organelle-specific DNP release. (A) Schematic representation of the organelle-specific release of DNP **12** for mitochondrial uncoupling in HeLa cells and cortical neurons. (B) TMRE indicates Δψ_m_ for mitochondrial uncoupling in HeLa cells. Scale bar: 10 μm. (C) TMRE indicates Δψ_m_ for mitochondrial uncoupling in cortical neurons. Scale bar: 10 μm. Arrows indicate cells expressing the mito-SNAP-FL protein. Quantification of TMRE fluorescence intensities of cells expressing mito-SNAP-FL protein shown at the bottom. (D) Cell-type-specific modulation of cortical neurons instead of glia cells. Scale bar: 10 μm. The statistical significance of the differences between groups was evaluated with the unpaired Student’s *t* test. The *p* values were calculated with the experiment group with light and caged-DNP **13**. A *p* value of 0.05 and below was considered to be significant: *p* < 0.01 (**) and *p* < 0.0001 (****). Data are presented as the mean ± SEM.

Mitochondrial uncoupler 2,4-dinitrophenols (DNP) **12** induces mitochondrial depolarization by binding with uncoupling protein 1 (UCP1); however, DNP also has many other cellular targets.^50,51^ The off-target effects result in obvious toxicity in clinical use, which calls for the development of controlled-release technology for DNP.^52^ We expect that the organelle-selective DNP release by selective photocatalysis may circumvent the side effects resulting from other cellular pathways, which hold promise for the targeted therapies (Figure 4A).^53,54^ We then synthesized the boronate-caged DNP **13** from DNP in one step and verified the boronate-caged DNP **13** uncaging by SNAP-FL protein in a cell-free system (Table S4, Figure S3D). Following the photocatalytic deboronative hydroxylation, caged DNP **13** converts to free DNP and quinone methides (QM). QM may hydrolyze to *p*-hydroxybenzyl alcohol by nucleophilic water addition or may be quenched by the physiological GSH to form QM-glutathione conjugates (Figure S9). HeLa cells expressing whole-cell-distributed SNAP-FL proteins were first constructed. Using TMRE as the mitochondrial membrane potential (Δψ_m_) indicator, boronate-caged DNP **13** incubation and green light irradiation resulted in no mitochondrial depolarization (Figure S10A). In sharp contrast, HeLa cells expressing mitochondria-localized mito-SNAP-FL protein exhibited evident mitochondrial depolarization under the identical optochemical treatment (Figure 4B, Figure S10B).

Neurons have complex cellular structures with ultrahigh sensitivity, which poses significant challenges but demonstrates more relevance to the therapeutic applications.^55^ The cultured mouse cortical neurons were then transfected with the mito-SNAP gene and treated with CLPDF **7**. In the cortical neurons expressing mito-SNAP-FL protein, mitochondria-selective DNP release resulted in mitochondrial depolarization (Figure 4C). It is worth noting the cell-type-specific construction of mito-SNAP-FL protein results only in mitochondrial depolarization in targeting neurons, while the neighboring glial cells were not affected. These results highlighted the cell-type-specific neuronal modulation (Figure 4D). Moreover, the neuron morphology was not altered, which indicated that the controlled generation of superoxide radical anions by photocatalysis is compatible with neurons (Figure S10C).^56^

### Dual Optogenetic/Optochemical Modulation of Synaptic Transmission with Projection Specificity

**Figure 5:**
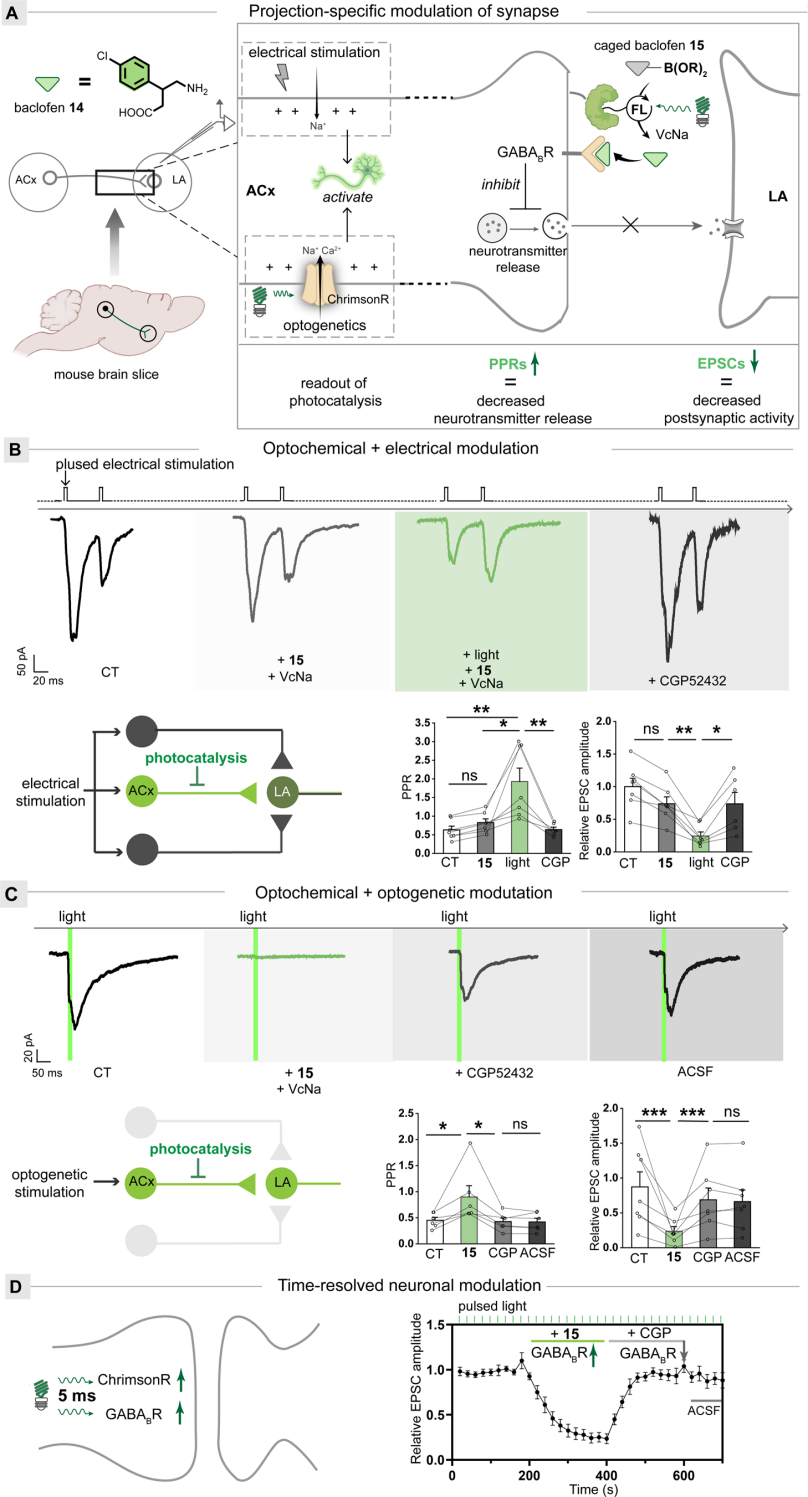
Projection-specific modulation of synaptic transmission with dual optogenetic/optochemical methods. (A) Schematic representation of EPSCs and PPRs changes by the photocatalytic uncaging of baclofen **14** within the ACx-LA synaptic projection in the brain. Electrical or optogenetic stimulation evoked EPSCs, when the optochemical uncaging of baclofen **14** was achieved using sodium ascorbates (VcNa) as reductants, activating presynaptic GABA_B_Rs to increase PPRs and decrease EPSCs. (B) Representative traces of EPSCs at ACx → LA synapses after electrical stimulation, caged **15** addition, optochemical uncaging, and CGP52432 (upper). Schematic projection representation of electrical stimulation and optochemical uncaging (bottom left). Histograms of mean ± SEM with circles denoting EPSC and PPRs (bottom right). *n* = 7 neurons from three C57 mice. (C) Representative traces of EPSCs at ACx → LA synapses after optogenetic stimulation, optochemical uncaging, CGP52432, and an artificial cerebrospinal fluid (ACSF) wash (upper). Schematic projection representation of optogenetic stimulation and optochemical uncaging (bottom left). Histograms of mean ± SEM with circles denoting EPSC and PPRs (bottom right). *n* = 7 neurons from three C57 mice. (D) Schematic representation of dual optogenetic/optochemical modulation (left). Time-resolved neuronal modulation at ACx → LA synapses with optogenetics and dual optogenetic/optochemical modulation (right, relative to baseline). The statistical significance of differences between groups was evaluated with the paired Student’s *t* test. A *p* value of 0.05 and below was considered significant: *p* < 0.05 (*), *p* < 0.01 (**), and *p* < 0.001 (***); ns is not statistically significant. Data are presented as the mean ± SEM.

Synapses generate and transmit cross-neuronal signals as subcellular structures of neurons, in which specific modulation in the long-range projections is desirable but remains challenging for neuroscience studies. Current optochemical methods could not achieve the projection-specific modulation of synaptic transmission due to the poor spatial precision of uncaged molecules in the illumination area.^57^ We expect that the photocatalytic modulation may elucidate the long-range synaptic projections of the B-type GABA receptor (GABA_B_R) (Figure 5A). The boronate-caged baclofen **15** was then prepared from GABA_B_R agonist baclofen **14** in one step and subjected to the cell-free system with SNAP-FL protein, which resulted in the smooth uncaging for baclofen **14** (Figure S2E, Table S4).^21^ We next fused a membrane-anchored extracellularly oriented SNAP-tag and an intracellularly localized mCherry to construct the SNAP-mCherry gene. The SNAP-mCherry gene was next transduced in the auditory cortex (ACx) of mice for the projection-specific synaptic investigation (Figure S11). After the mice were sacrificed, the brain slices containing the ACx-innervated lateral amygdala (LA) region were incubated with BGFL **1** to construct the SNAP-FL protein at the ACx-to-LA projection within corticoamygdalar circuits.

The electrophysiology was then used to assess GABA_B_R activation by measuring paired-pulse ratios (PPRs) and excitatory postsynaptic currents (EPSCs). The PPRs’ increase demonstrates the decreased presynaptic neurotransmitter release, while the EPSCs’ decrease indicates the baclofen **14** uncaging to decrease postsynaptic activity. In the control group with only boronate-caged baclofen **15** incubation, the amplitude of PPRs or EPSCs was not changed (Figure 5B). In contrast, the experimental group with green light irradiation for several minutes resulted in a significant increase in PPRs and a decrease in EPSCs. Afterward, the addition of GABA_B_R inhibitor CGP52432 completely abolished these effects, which further confirmed the selective GABA_B_R activation by baclofen **14**.

In contrast to electrical stimulation unbiasedly targeting multiple projections, the optogenetic methods can aim the single projection accurately.^58^ For the optogenetic modulation, we transduced the photosensitive ion channel ChrimsonR at the ACx-to-LA projection. For the optochemical modulation, we transduced SNAP-mCherry at the ACx-to-LA projection for dual photomodulation of synaptic responses (Figure S11). Under basal conditions (CT) with optogenetic stimulation alone, repetitive light pulses of 5 ms duration evoked stable EPSCs and PPRs in the ACx-to-LA projection (Figure 5C). After treatment with caged baclofen **15**, the repetitive light pulses of 5 ms duration photocatalytically uncaged baclofen **14** and evoked dual optogenetic/optochemical modulation, which was shown as a progressive increase in PPRs and a decrease in EPSCs (Figure 5D). The efficiency achieved by optochemical methods is remarkable in that one 5 ms light pulse suffices for the EPSC inhibition, which has not been achieved by other methods. Five repetitive light pulses with a total duration of 25 ms completely inhibited the EPSC.

As the photocatalytic uncaging of baclofen **14** has projection specificity, only GABA_B_Rs at this ACx-to-LA projection within corticoamygdalar circuits could be activated. For electrical stimulation, multiple corticoamygdalar projections are activated. As a result, baclofen **14** uncaging at a single ACx-to-LA projection could not completely inhibit the EPSCs. In contrast, the optogenetic activation of EPSCs could be completely inhibited by baclofen **14** uncaging at the ACx-to-LA projection in some cases. In those cases, the EPSCs at a single ACx-to-LA projection are activated by optogenetics, which results in EPSC being completely inhibited by baclofen **14** uncaging at this very projection. Afterward, the addition of CGP52432 completely abolished the optochemical modulation to recover EPSCs, while the optogenetic stimulation was not reversed. We calculated the concentration of baclofen generated by 5 ms pulse light excitation to be approximately 0.46 μM, a value closely approaching the minimum concentration required for GABA_B_R activation (approximately ∼0.1–0.5 μM).^59^ Due to the free diffusion of uncaged baclofen, its concentration in untargeted sites drops sharply to limit the GABA_B_R activation to the ACx-to-LA projection (Figure S12). This result suggests that photocontrolled uncaged molecules exhibit high projection specificity even with free diffusion in extracellular compartments. The spatial precision provided by this method surpasses that of tumor-specific click-to-release methods for better dose control.^60^ Together, these results highlight the potential of dual optogenetic/optochemical methods in dissecting distinct signaling pathways in a single projection.^61^

### Cell-Type-Specific Baclofen Release Dissects an Anti-Itch Signaling Mechanism in Live Mice

**Figure 6:**
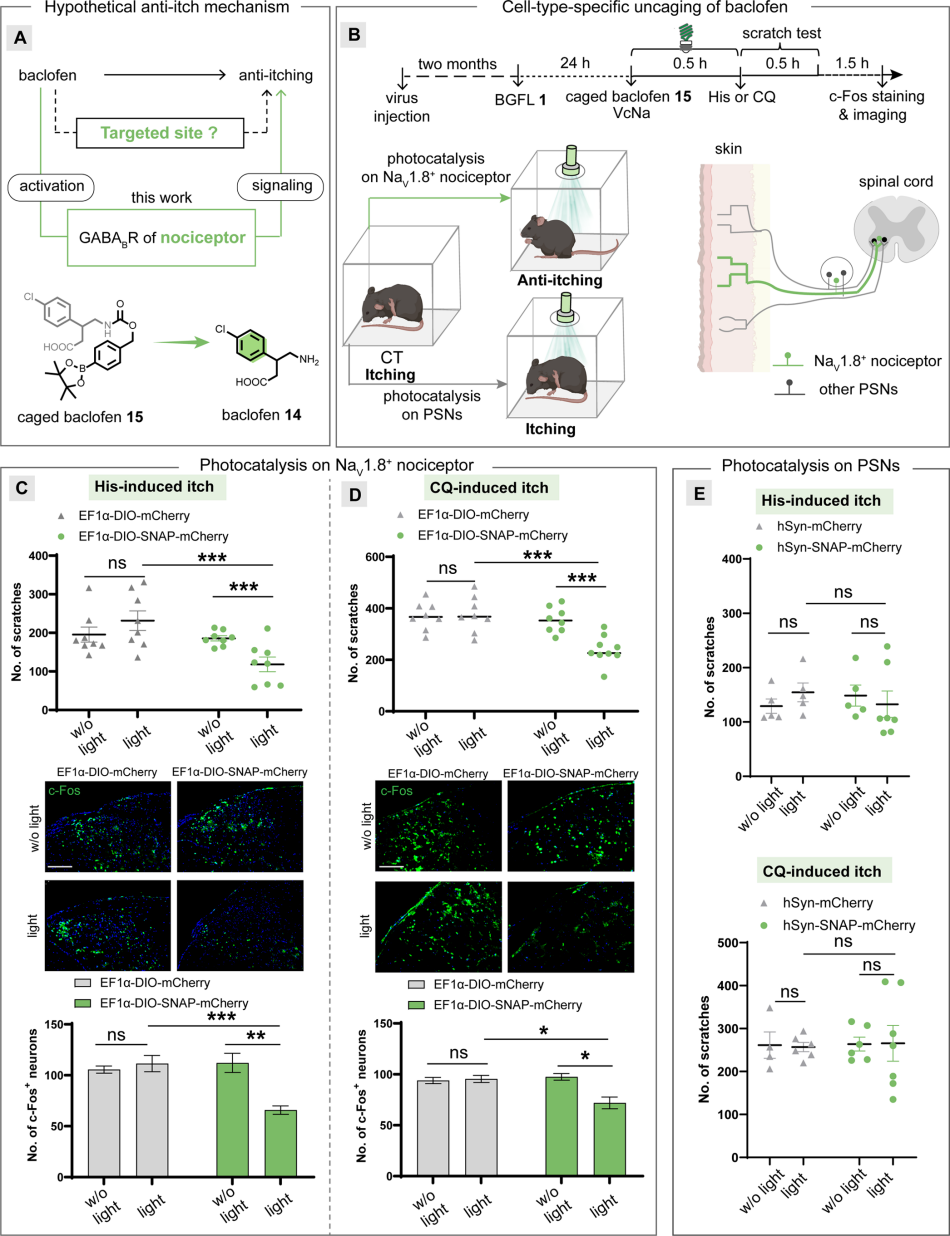
Anti-itch signaling mechanism dissection in live mice with cell-type-specific baclofen release. (A) Hypothetical anti-itch signaling mechanism by baclofen **14**. (B) Experimental procedure of inhibiting the Na_V_ 1.8^+^ nociceptor or other PSNs for antipruritic signal modulation. The optochemical release of baclofen **14** on the Na_V_ 1.8^+^ nociceptor inhibited acute itch induced by histamine (His) or chloroquine (CQ) in mice. (C) Antipruritic effect of baclofen **14** release on the Na_V_ 1.8^+^ nociceptor against His-induced itch, which was characterized by the number of scratches (*n* = 6) (upper) and neurons expressing c-Fos (*n* = 3) (bottom). Representative images of the spinal cord with c-Fos staining (middle). Scale bar: 100 μm. (D) Antipruritic effect of baclofen **14** release on the Na_V_ 1.8^+^ nociceptor against CQ-induced itch, which was characterized by the number of scratches (*n* = 6) (upper) and neurons expressing c-Fos (*n* = 3) (bottom). Representative images of the spinal cord with c-Fos staining (middle). Scale bar: 100 μm. (E) No antipruritic effect of baclofen **14** release against His- (upper) or CQ- (bottom) induced itch on pan PSNs, which was characterized by the number of scratches (*n* = 6). The statistical significance of the differences between groups was evaluated with the unpaired Student’s *t* test. A *p* value of 0.05 and below was considered to be significant: *p***<** 0.05 (*), *p***<** 0.01 (**), and *p***<** 0.001 (***); ns is not statistically significant. Data are presented as the mean ± SEM.

Inhibitory GABAergic transmission on sensory neurons is a key neurological pathway to suppressing itch sensation.^62,63^ Among those, GABA_B_R is widely studied as a major receptor type for GABA.^64^ However, the precise site for GABA_B_R activation within various peripheral afferent sensory terminals remains unknown, as there is no specific method to activate receptors on certain cell types. We were curious to know if the cell-type-specific optochemical activation of GABA_B_R may decipher such antipruritic signaling (Figure 6A). The prescription drug baclofen **14** can alleviate acute itch induced by histamine (His) or chloroquine (CQ).^64^ Our initial experiments verified that the subcutaneous injection of baclofen **14** significantly reduced the number of scratches of mice. On the molecular level, the c-Fos expression in the superficial laminae of the dorsal spinal cord was significantly reduced as the neuronal activity marker (Figure S13A,B).

The boronate-caged baclofen **15** was then subcutaneously injected into live mice together with small-molecule fluorescein, followed by green light irradiation of the skin surface. The photocatalytic generation of baclofen **14** by fluorescein performed well under the skin in live mice, which represents the first photocatalytic modulation of neuronal activity in live mice by visible light irradiation. The reduction of the number of scratches and c-Fos expression in the dorsal spinal cord indicated the photocatalytic GABA_B_R activation in sensory terminals innervating the skin (Figure S13C). However, small-molecule fluorescein photocatalysis could not achieve the cell-type-specific release of baclofen **14** by the restricted uncaging of boronate-caged baclofen **15**. We next evaluated if the antipruritic effect was resulted from the Na_V_1.8-expressing nociceptors exclusively or across all sensory terminals (Figure 6B).^65,66^ To this end, the cell-type-specific activation of GABA_B_R by the genetically encoded photocatalysis uniquely suits this need.

The Cre-inducible AAV (AAV-EF1α-DIO-SNAP-mCherry) was employed to transduce the SNAP-mCherry gene into the peripheral nervous system with the AAV-PHP.S capsid in Na_V_1.8-Cre mice (Figure S13D).^67^ BGFL **1** was next subcutaneously injected for 1 day to construct the SNAP-FL protein specifically in Na_V_1.8^+^ nociceptors in live mice. A low-dose caged baclofen **15** injection was then performed on live mice, followed by the treatment with His or CQ. After a 30 min green light illumination, the number of scratches induced by His or CQ was significantly decreased, which was attributed to the Na_V_1.8^+^ nociceptors-specific baclofen release. In addition, a significant reduction in the staining of c-Fos^+^ neurons was observed after sacrificing the mice.

In sharp contrast, mice-expressed mCherry proteins instead of SNAP-mCherry proteins showed no effect after the identical BGFL, caged baclofen **15**, and green light treatments (Figure 6C,D). Importantly, the unbiased expression of SNAP-mCherry proteins on peripheral sensory neurons (PSNs) resulted in no antipruritic effect after identical treatments (Figure 6E). These results together suggest that Na_V_1.8^+^ nociceptors, rather than pan PSNs, contribute to the GABA_B_R-mediated antipruritic effect. Such cell-type-specific modulation of receptor signaling highlights the significance of optochemical methods in revealing cell-type-specific signaling pathways, which remains unaccomplished by current methods.

## Conclusions

The genetically encoded photocatalysis method is developed to modulate neuronal activity by the metal-free deboronative hydroxylation in cultured cells, brain slices, and live mice. The genetically encoded photocatalysts uncage photoinsensitive organoboronates for versatile neuronal modulation with organelle, projection, and cell-type specificity. Doxorubicin, a mitochondrial uncoupler, and baclofen are photocatalytically released for organelle-targeted drug delivery, dual optogenetic/optochemical control of synaptic transmission, and anti-itch signaling mechanism dissection. The itch alleviation in live mice is revealed for the first time to be attributed to GABA_B_R activation in the Na_V_1.8-expressing nociceptor afferent projection instead of pan PSNs.

## Supporting Information Available

Mechanistic studies, uncaging reactions *in**vitro*, genetic construct, mammalian cell studies, neuronal studies, substrate preparations and characterizations, and NMR spectra of new compounds (PDF)
