# Supplementary material for: Genetically Encoded Photocatalysis Enables Spatially Restricted Optochemical Modulation of Neurons in Live Mice
Source: ACS Cent Sci. 2023 Dec 18;10(1):163–75. doi: 10.1021/acscentsci.3c01351 (PMC10823520; doi:10.1021/acscentsci.3c01351)
Supplement: Supplementary file 1 — oc3c01351_si_001.pdf [file oc3c01351_si_001.pdf]

Genetically Encoded Photocatalysis Enables Spatially Restricted Optochemical Modulation of Neurons in Live Mice

Supplementary Information

Kaixing Zeng[1,2#], Zhi-Han Jiao[3#], Qin Jiang[3], Ru He[1,2], Yixin Zhang[1], Wei-Guang Li[3,4]*, Tian-Le Xu[3]*, and Yiyun Chen[1,2,5]*

[1]State Key Laboratory of Chemical Biology, Shanghai Institute of Organic Chemistry, University of Chinese Academy of Sciences, Chinese Academy of Sciences, 345 Lingling Road, Shanghai 200032 China. [2]School of Physical Science and Technology, ShanghaiTech University, 100 Haike Road, Shanghai 201210 China. [3]Centre for Brain Science and Department of Anatomy and Physiology, Shanghai Jiao Tong University School of Medicine, 280 South Chongqing Road, Shanghai 200025, China. [4]Department of Rehabilitation Medicine, Huashan Hospital, Institute for Translational Brain Research, State Key Laboratory of Medical Neurobiology and Ministry of Education Frontiers Centre for Brain Science, Fudan University, 131 Dongan Road, Shanghai 200032, China. [5]School of Chemistry and Material Sciences, Hangzhou Institute for Advanced Study, University of Chinese Academy of Sciences, 1 Sub-lane Xiangshan, Hangzhou 310024, China

*Professor Wei-Guang Li
Email: liwg@fudan.edu.cn

*Professor Tian-Le Xu
Email: xu-happiness@shsmu.edu.cn

*Professor Yiyun Chen
Email: yiyunchen@sioc.ac.cn
http://yiyunchen.sioc.ac.cn

Table of Contents

1. Methods..3
2. Supplementary Figures and Tables 12
3. Genetic Construct..27
   Plasmid..27
   Virus..28
4. Synthesis of Small Molecules..29
5. NMR Spectra of New Compounds 35
6. References..39

1. Methods

General methods and materials

Unless otherwise noted, all reactions of substrate preparation were conducted in flame-dried glassware under a nitrogen atmosphere using an anhydrous solvent passed through an activated alumina column (*Innovative Technology*). Commercially available reagents were used without further purification. Thin layer chromatography (TLC) was performed using Jiangyou TLC silica gel plates HSG F_{254} and visualized using UV light and potassium permanganate. Flash chromatography was performed on Lisure Science EZ purification system using the Santai technologies silica gel cartridge. ^{1}H and ^{13}C NMR spectra were recorded on an Agilent 500 MHz spectrometer. Chemical shifts in ^{1}H NMR spectra were reported in parts per million (ppm) on the δ scale from an internal standard of residual $CDCl_3$ (7.26 ppm), CD_3OD (3.31 ppm) or DMSO-d_6 (2.50 ppm). Data for ^{1}H NMR were reported as follows: chemical shift, multiplicity (s = singlet, d = doublet, t = triplet, q = quartet, dd = doublet of doublets, ddd = doublet of double doublets, m = multiplet, br = broad), coupling constant in Herts (Hz) and integration. Data for ^{13}C NMR spectra were reported in terms of chemical shift in ppm from the central peak of $CDCl_3$ (77.16 ppm), CD_3OD (49.00 ppm) or DMSO-d_6 (39.52 ppm). IR spectra were recorded on a Thermo Scientific Nicolet 380 FT-IR spectrometer. MS experiments were performed on a Bruker maXis 4G instrument for HRMS-ESI. BGAF was synthesized as previously described in the literature.[1-2] Boronate-caged coumarin **3**, ADPA, and boronate-caged baclofen **15** were synthesized in our previous work.[3] The synthetic procedures of other compounds are in Supplementary Methods Online. Analytical HPLC was conducted on a Dionex controller by using a C_{18} 4.6 nm×250 mm reverse phase column with UV detection. The light intensity was measured with an International Light ILT1400 photometer equipped with SEL033/QNDS2/W photodetector (2.5 cm from 4 W green LED: 2.9 mW/cm^2; 0.5 cm from 4 W green LED: 10 mW/cm^2; 0.5 cm from 35 W green LED: 95 mW/cm^2; 15.0 cm from the Xe light at 500 nm: 4.5 mW/cm^2). ChemiDocTMXRS+ gel documentation and analysis systems (Bio-Rad) recorded fluorescence images and coomassie blue-stained protein bands. The absorption was measured by Nanodrop 2000c UV/Vis spectrometer. The fluorescence intensity was measured with SpectraMax i3x Multi-Mode Microplate Reader (Molecular Devices). All animal procedures were approved by the Animal Ethics Committee of Shanghai Jiao Tong University School of Medicine and by the Institutional Animal Care and Use Committee (Department of Laboratory Animal Science, Shanghai Jiao Tong University School of Medicine; Policy Number DLAS-MP-ANIM. 01–05).

Protein Expression and Purification

The genes encoding SNAP were cloned into the expression plasmids pSJ2 with an N terminal fusion peptide containing 8×His affinity tag and TEV protease recognition and cleavage site (MGSHHHHHHHHGSDYDIPTTENLYFQGS). The resulting expression vectors pSJ2 were transformed into BL21(DE3) (TIANGEN). Cell culture was grown to an optical density at 600 nm (OD_{600}) of 0.6-0.8 in Luria–Bertani (LB) medium at 37 °C. SNAP protein was expressed by adding isopropyl-β-D-thiogalactoside (IPTG; final concentration 0.3 mM) at 16 °C, after which the E. coli cells were harvested by centrifugation. The proteins were purified with Ni–NTA agarose (Cytiva). The purified proteins were stored in aliquots in the storage buffer (50 mM Tris-Cl, 250 mM NaCl, 4 mM DTT, 0.1 mM EDTA, pH 7.5) at –80 °C with 10% glycerol.

Photocatalytic boronate uncaging reactions by SNAP-FL in the cell-free system

Unless otherwise noted, all reactions were conducted in PBS buffer (10 mM, pH 7.4) in 0.2 mL colorless EP tubes. BGFL (1.0 eq.) and SNAP-tag (1.0 eq.) were mixed in 0.2 mL PBS buffer (10 mM, pH 7.4) and incubated for 60 seconds at room temperature to generate SNAP-FL. After SNAP-FL was constructed, boronate-caged substrates and reductants (NADH or ascorbates) were added. Reactions were carried with 516 nm green LEDs (2.9 mW/cm^2). The irradiated condition for light sources was 2.5 cm from the green LED. Afterward, a mixture sample was treated in ultrafiltration tubes to exclude the proteins (12, 000 r.p.m, 30 min, 4 °C). Then the mixture (50 µL) was subjected to HPLC analysis to afford conversions and yields.

Photocatalytic oxidation of NADH

NADH (50 µM) and 1 µM FL or SNAP-FL were mixed in 1.0 mL PBS buffer (10 mM, pH 7.4). The mixture was added into the cuvette and irradiated for different times at λ = 500 nm (a smart xenon lamp light source, 4.5 mW /cm^2) under air. The absorbance spectra were measured immediately after each irradiation. The concentration of NADH was determined by absorbance at 334 nm.

Superoxide radical anion detection

Superoxide radical was detected with dihydrorhodamine 123 (DHR 123) according to literature methods.[4] A solution of 5 µM SNAP-FL, 1 mM NADH and DHR 123 (100 µM) in 25 µL PBS buffer (10 mM, pH 7.4) was added to 0.2 mL PCR tube. Then samples were irradiated with 4 W green LED light (5.5 cm from LED, 516 nm, 2.9 mW/cm^2) under air atmosphere at ambient temperature. The fluorescence intensity was measured (Ex = 480 nm, Em = 530 nm).

Singlet oxygen detection

Singlet oxygen was detected with 9,10-anthracenedipropanoic acid (ADPA) according to literature methods.[5] Reductant (100 μM), FL or SNAP-FL (10 μM), and ADPA (200 μM) were mixed in PBS buffer (10 mM, pH 7.4) at ambient temperature. The samples were added to 1 mL cuvette and irradiated with a Xe light (15.0 cm from the Xe light, 500 nm, 4.5 mW/cm^2) under an air atmosphere. The absorption of the samples irradiated for different times was measured, and the absorbance at 400 nm was used for calculation.

Estimation of the diffusion radius of superoxide radical anion

The diffusion radius of superoxide radical anion ($O_2^{\cdot-}$) is estimated using Fick's law,[6] as shown in Supplementary equation (1).

$$\Delta x = -\frac{\ln\left(\frac{[O_2^{\cdot-}]}{[O_2^{\cdot-}]_0}\right)}{\sqrt{\frac{ln2}{Dt_{1/2}}}} \quad (1)$$

where Δx is the diffusion radius, D is the diffusion coefficient, and $t_{1/2}$ is the half-life for $O_2^{\cdot-}$. The distance over which the $O_2^{\cdot-}$ concentration $[O_2^{\cdot-}]$ drops to a tenth, that is $[O_2^{\cdot-}]/[O_2^{\cdot-}]_0 = 0.1$, is taken as its Δx. D refers to that of $O_2^{\cdot-}$ in the water and is about 1000 μm^2 s^{-1}.[7] The $t_{1/2}$ of $O_2^{\cdot-}$ in biological systems was estimated in the presence of superoxide dismutase (SOD), following a pseudo-first-order reaction equation (Supplementary equation (2)).

$$t_{1/2} = \frac{ln2}{k\{SOD\}_0} \quad (2)$$

k is the second-order decay rate constant of $O_2^{\cdot-}$ with SOD and is more than 10^9 M^{-1}s^{-1}.[8] $[SOD]_0$ is a nominal cellular SOD concentration of about 100 μM.[9] According to the equations (1) and (2), the diffusion radius of $O_2^{\cdot-}$ in biological systems was estimated as 0.23 μm.

For singlet oxygen ($^1O_2$), its diffusion radius was estimated as 70 nm.[10]

Intracellular SNAP-FL construction in cells

Cells were seeded in the appropriate dishes or plates in the culture medium [Fetal Bovin Serum, South American, S1001-500] (BIOAGRIO, Co., LTD.), and were then transfected with the SNAP fusion construct of interest using PolyJet™ (SignaGen Laboratories). Typically, plasmid and PolyJet™ in DMEM (without serum and antibiotics) were used for cells in each well in a 24-well plate. After 24 h transfection, 2 μM CLPDF **7** was added to the media and incubated for 2 hours. Cells were washed with DPBS three times, then the localized fluorescence of SNAP-FL could be observed by fluorescent microscopy.

Photocatalytic uncaging of boronate-caged aminocoumarin in HeLa cells

Cells were seeded in a 24-well plate with a glass coverslip in the culture media. After constructing intracellular SNAP-FL, cells were incubated in the fresh DMEM (no phenol red) containing 20 μM caged aminocoumarin **8** and 0.5 mM NADH for 30 min. The cell culture was irradiated with the 520 nm green LED (95 mW/cm^2) for 15 min at room temperature under an air atmosphere. For control experiments, cells were added with the same amount of DMSO instead of CLPDF, cells were added DMEM without transfection reagent and plasmid (no SNAP group), or cells were kept in the dark during light irradiation. After photocatalytic uncaging, cells were washed three times with DPBS, then fixed with 4% paraformaldehyde in PBS at room temperature for 15 minutes. Samples were washed with PBS before being mounted with Fluorescent Mounting Medium (*Dako*) and sealed with nail polish. Images were acquired on a Leica TCS SP8 confocal laser scanning microscopy platform with 63× oil-immersion objective lens by using the following regular settings: 405 nm laser with DAPI filter (Em = 415-485nm), 488 nm laser with FICT filter (Em = 495-530 nm) and 561 nm laser with TRITC filter (Em=575-615 nm). Digital pictures of different samples in each group were taken under identical conditions of gain and exposure, and the pictures in different channels were merged using ImageJ. The colors of the DAPI and FITC channels were green and red, respectively. Pearson's correlation coefficient (*R*) was calculated using Coloc 2 plugin in ImageJ.

Photocatalytic uncaging of boronate-caged DOX in HeLa cells

Cells were seeded in a 48-well plate in the culture media. After constructing intracellular SNAP-FL, cells were incubated in the Hank's balanced salt solution (HBSS) containing 20 μM caged DOX **11** and 1 mM NADH for 30 min. The cell culture was irradiated with the 520 nm green LED (10 mW/cm^2) for 10 min at room temperature under an air atmosphere. For control experiments, cells were added with the same amount of DMSO instead of caged DOX **11**, cells were added DMEM without transfection reagent and plasmid (w/o SNAP group), or cells were kept in the dark during light irradiation. After the photocatalytic uncaging reaction, HBSS was replaced with fresh DMEM (+10% FBS), and cells were cultured for 12 h. MTT reagent were added to each well and incubated for 2 h. The produced formazan was dissolved in DMSO, and the absorbance at 570 nm was measured with a Spectramax Microwell plate reader (Molecular Devices). The background absorbance was measured and subtracted at 690 nm. The cell viability was calculated as Abs_{570}-Abs_{690}. The cell viability of cells treated with DMSO alone was used as a 100% standard in normalization.

Photocatalytic uncaging of boronate-caged DNP in HeLa cells

After constructing mito-SNAP-FL inside the cell, neurons were incubated in the fresh DMEM (no phenol red) containing 100 μM caged DNP **13** and 2 mM VcNa for 1 h. The cell culture was irradiated with the 520 nm green LED (10 mW/cm^2) for 10 min at room temperature under air atmosphere, then was incubated for 30 min in fresh DMEM followed by treatment with TMRE (5 μM) for 30 min and after once wash, the live cells were observed on the confocal laser scanning microscope. In control groups, cells were added without caged DNP, without transfection, transfected with SNAP instead of mito-SNAP, and kept in the dark during light irradiation. Images were acquired on a Leica TCS SP8 confocal laser scanning microscopy platform with 40× dry objective lens by using the following regular settings: 488 nm laser with FICT filter (Em = 495-530 nm) and 561 nm laser with TRITC filter (Em = 575-615 nm). Digital pictures of different samples in each group were taken under identical conditions of gain and exposure, and the pictures in different channels were merged using ImageJ. The fluorescence was calculated with Image J.

Photocatalytic uncaging of boronate-caged DNP in cortical neurons

The pregnant mice from 17 to 18 days of pregnancy were killed with ether anesthesia, sterilized in 75% alcohol for a moment, cut open the exposed abdominal cavity and taken out the Y-shaped uterus containing the fetal mice, and placed in a sterile Petri dish. The uterus was cut on the ice plate in the ultraclean table, and the head of the fetal rat was successively cut by the Surgical scissors and placed in a sterile Petri dish containing precooled commercial D-Hanks solution (containing 1% v/v penicillin + Streptomycin, 0.1% v/v HEPES solution). The whole brain of the fetal rat was taken out in the Petri dish containing D-Hanks solution, transferred to another Petri dish containing the precooled D-Hanks solution, and isolated cerebral cortex. Then it was rinsed with the precooled D-Hanks solution mentioned above, transferred to the separated cortical tissue into EP tube, added commercial 0.25% trypsin, shaken gently and evenly, and digested in a 37 °C incubator. After digestion is completed, transfer to the whole culture high sugar DMEM medium to terminate digestion. Put the suspension into a new Petri dish with a cell filter screen, and centrifugate the supernatant. Resuspension with full culture high sugar DMEM medium and seed into cultivation dish. After 24 hours of cultivation, cells were washed twice with PBS or DMEM, and the entire culture medium was replaced with a Neurobasal culture medium (containing 2% v/v B27 and 1% v/v Glutamax) to continue cultivation. Neurons were transfected at DIV7–10 (7-10 days in vitro). For transfection, the original medium was changed to a fresh Neurobasal medium. In a 35-mm

dish, 1 μg–4 μg plasmid of mito-SNAP was mixed with 60 μL $CaCl_2$ (0.3 mol/L) by pipetting up and down, then HBSS was added. After thoroughly mixing, the transfection solution was immediately transferred into the dish with neurons. After incubation at 37°C for 1 h-1.5 h, the medium was replaced with Neurobasal medium (wash medium) to remove excess calcium phosphate particles. After that, the wash medium was replaced with the original medium, and fresh Neurobasal medium with B27 supplement. The dish was then returned to the culture incubator.

At 18 DIV (18 days in vitro), photocatalytic uncaging and live cell imaging experiments were performed. Neurons were treated with CLPDF **5** for 2 h followed by wash for three times. After constructing intracellular mito-SNAP-FL, neurons were incubated in the fresh medium (no phenol red) containing 30 μM caged DNP and 1 mM VcNa for 30 min. The cell culture was irradiated with the 520 nm green LED (4 W) for 4 min at room temperature under an air atmosphere, then treated with TMRE (5 μM) for 30 min. After washing, the live cells were observed on the confocal laser scanning microscope. In control groups, cells were added without caged DNP or kept in the dark during light irradiation. Images were acquired on a confocal laser scanning microscopy (Digital Eclipse A1R+, Nikon) platform with 63× oil-immersion objective lens by using the following regular settings: 488 nm laser with FICT filter (Em=495-530 nm) and 561 nm laser with TRITC filter (Em=575-615 nm). Digital pictures of different samples in each group were taken under identical conditions of gain and exposure.

Virus injection

Mice at 6–7 weeks old were anesthetized with 1% sodium pentobarbital via a single intraperitoneal injection (10 ml per kg of body weight), after which each mouse was mounted in a stereotactic frame with non rupture ear bars (RWD Life Science, Shenzhen, China). After making an incision to the midline of the scalp, small bilateral craniotomies were performed using a microdrill with 0.5-mm burrs. Glass pipettes (tip diameter: 10–20 μm) were made with a P-97 Micropipette Puller (Sutter glass pipettes, Sutter Instrument Company, USA) for AAV microinjections. The microinjection pipettes were filled with silicone oil and then connected to a microinjector pump (RWD Life Science, Shenzhen, China) to achieve complete air exclusion.

For assays in brain slices, AAV-containing solutions were loaded into the tips of pipettes and injected at the following coordinates (anteroposterior to bregma, AP; lateral to the midline, ML; below the bregma, DV; in mm): ACx: AP; −2.6; ML, ±4.0; DV, −2.4. Virus-containing solutions were injected bilaterally/unilaterally into the ACx (0.3 μl/side), at a rate of 0.1 μL/min. After injection, the pipette was left in place for an additional 10 min to allow the injectant to

diffuse adequately. For itch-related assays, 100-150 μL per mouse of viral solution containing 5×10^{12} vg/mL was injected into the mice via the tail vein. Mice were allowed to recover for at least 3 weeks before behavioral and other tests, and the injection sites were examined at the end of the experiment by the expression of the fluorescent protein mCherry.

Slice electrophysiology

Whole-cell recordings were performed in acute brain slices from those that had been stereotaxically injected with AAV-CaMKIIα-SNAP-mCherry or AAV-Syn-ChrimsonR-EGFP in the same ACx region. Mice were deeply anesthetized with 1% sodium pentobarbital and were subsequently decapitated. Brains were dissected quickly and were chilled in well-oxygenated (95% O_2/5% CO_2, v/v) ice-cold artificial cerebrospinal fluid (ACSF) containing the following (in mM): 125 NaCl, 2.5 KCl, 12.5 D-glucose, 1 $MgCl_2$, 2 $CaCl_2$, 1.25 NaH_2PO_4, and 25 $NaHCO_3$ (pH 7.35-7.45). Coronal brain slices (300-μm thick) containing regions of interest were cut with a vibratome (Leica VT1000S, Germany). After recovery for 1 hour in oxygenated ACSF at 30 ± 1 °C, each slice was transferred to a recording chamber and was continuously superfused with oxygenated ACSF at the rate of 1–2 mL per minute. The neurons in LA were patched under visual guidance using infrared differential-interference contrast microscopy (BX51WI, Olympus) and an optiMOS camera (QImaging). During all electrophysiological studies, the slices were continuously perfused with well-oxygenated ACSF at 35 ± 1 °C. Whole-cell patch-clamp recordings were performed using an Axon 200B amplifier (Molecular Devices). Membranous currents were sampled and analyzed using a Digidata 1440 interface and a personal computer running Clampex and Clampfit software (Version 10, Axon Instruments). Access resistance was 15-20 MΩ, and only cells with a change in access resistance < 20% were included in the analysis. The brain slices were treated with boronate-caged baclofen **15** (20 μM) and VcNa (40 μM) at indicated time point. Photocatalytic uncaging was performed using a collimated LED (Lumen Dynamics) with peak wavelengths of 532 nm. The LED was connected to an Axon 200B amplifier to trigger photo stimulation. The brain slice in the recording chamber was illuminated through a 40 × water-immersion objective lens (LUMPLFLN 40XW, Olympus). The intensity of photo-stimulation was directly controlled by the stimulator (1800 mW/cm^2), while the duration was set through Digidata 1440 and pClamp 10.5 software.

Light-evoked EPSCs: Each slice was illuminated every 20 s with green light pulses of 5-ms durations to evoke synaptic responses in the LA by optogenetic photo-stimulation of ACx axons. To prevent polysynaptic activities from being detected in EPSC recordings, the

appropriate photo-stimulation intensities were applied that produced 30–50% of the maximal synaptic response. For recording light-evoked EPSCs, the recording pipettes (3–5 MΩ) were filled with a solution containing the following (in mM): 132.5 cesium gluconate, 17.5 CsCl, 2 $MgCl_2$, 0.5 EGTA, 10 HEPES, 4 Mg-ATP, and 5 QX-314 chloride (280–300 mOsm, pH 7.2 with CsOH). To determine the paired-pulse ratio (PPR), the patched LA neurons were voltage clamped at –70 mV. The AMPAR EPSCs were evoked by paired photo-stimulations (20-ms intervals; 5-ms duration) of opsin-expressing axons, and PPRs were calculated as the peak amplitude ratio of the second to the first EPSC. Then $GABA_BR$ antagonist CGP52432 (20 μM) was added to the fluid to block the $GABA_BR$.

Electrical stimulation-evoked EPSCs: EPSCs were recorded from LA principal neurons with an Axon 200B amplifier (Molecular Devices), and the stimulations were delivered with a bipolar tungsten stimulating electrode (0.1-ms duration) placed on the fibers entering in the external capsule to stimulate cortical glutamatergic inputs to the LA. The AMPAR-mediated EPSCs were induced by repetitive stimulations at 0.05 Hz, with the patched neuron voltage clamped at −70 mV. The patched LA neurons were voltage clamped at –70 mV to determine the PPR.

Mice and itch-related behavioural assays

Three to four mice were housed per cage and maintained on a 12 hr light/dark cycle with food and water ad libitum. Mice were acclimatized for 30 min before all behavioral experiments. (1) Baclofen treatment: The saline solution of baclofen **14** (3.74 mM) was subcutaneously injected into the nape after acclimatization. After 30 min, pruritic compounds (histamine or chloroquine) were injected into the nape subcutaneously. (2) Nonspecific uncaging of baclofen by FL: the saline solution containing FL (0.90 mM), caged baclofen **15** (3.74 mM), and VcNa (7.48 mM) was subcutaneously injected into the nape after acclimatization followed by 30 min 516 nm green LED light irradiation (2.9 mW/cm^2). (3) This method: for SNAP-mCherry or mCherry expressing mice, 10 μM BGFL in saline was injected subcutaneously in mice. After one day, the saline solution containing caged baclofen **15** (500 μM), and VcNa (1 mM) was subcutaneously injected into the nape after acclimatization followed by 30 min 516 nm green LED light irradiation (2.9 mW/cm^2).

After reagent treatment and light irradiation, pruritic compounds (histamine or chloroquine) were subcutaneously injected into the nape, and scratching behaviors were observed for 30 min. A bout of scratching was defined as continuous scratch movements with hind paws directed at the area around the injection site. Scratching behavior was quantified by recording the number

of scratching bouts for the 30 min observation period. All the behavioral experiments were conducted and scored with the experimenter blinded to the genotype and the compound treatment.

Histology and fluorescent immunostaining

The spinal cord was separated from sacrificed mice 1.5 h immediately after the scratch test, and frozen spinal cord coronal slices were stained for c-Fos. Slides were imaged using an Olympus VS120 virtual microscopy (Olympus, Japan) slide-scanning system. All specimens were blinded with respect to genotype and treatment before imaging, and the number of c-Fos-positive (c-Fos$^+$) neurons in the dorsal horn was counted.

For the c-Fos staining, spinal cord coronal slices were washed three times (10 min each time) with1 × PBS and were then blocked with 10% normal donkey serum in 1 × PBS with 0.3% Triton X-100 (PBST) for 1 h, after which they were incubated overnight at 4°C with rabbit anti-c-Fos (1:500, Cell Signaling Technology, catalog no. 2250). Sections were then washed with PBS, incubated in 2% normal donkey serum for 10 min, and then incubated for 2 h with Alexa Fluor® 568 donkey anti-rabbit IgG (H+L) (ThermoFisher Scientific, catalog no. A10042). Sections were washed in 1 × PBS with 0.1% Tween-20, mounted onto slides, and cover slipped with ProLong Gold Antifade Mountant(Invitrogen). Quantification was performed by counting the number of c-Fos$^+$ cells in the dorsal horn of the spinal cord. All counts were performed blind with respect to treatment groups.

2. Supplementary Figures and Tables

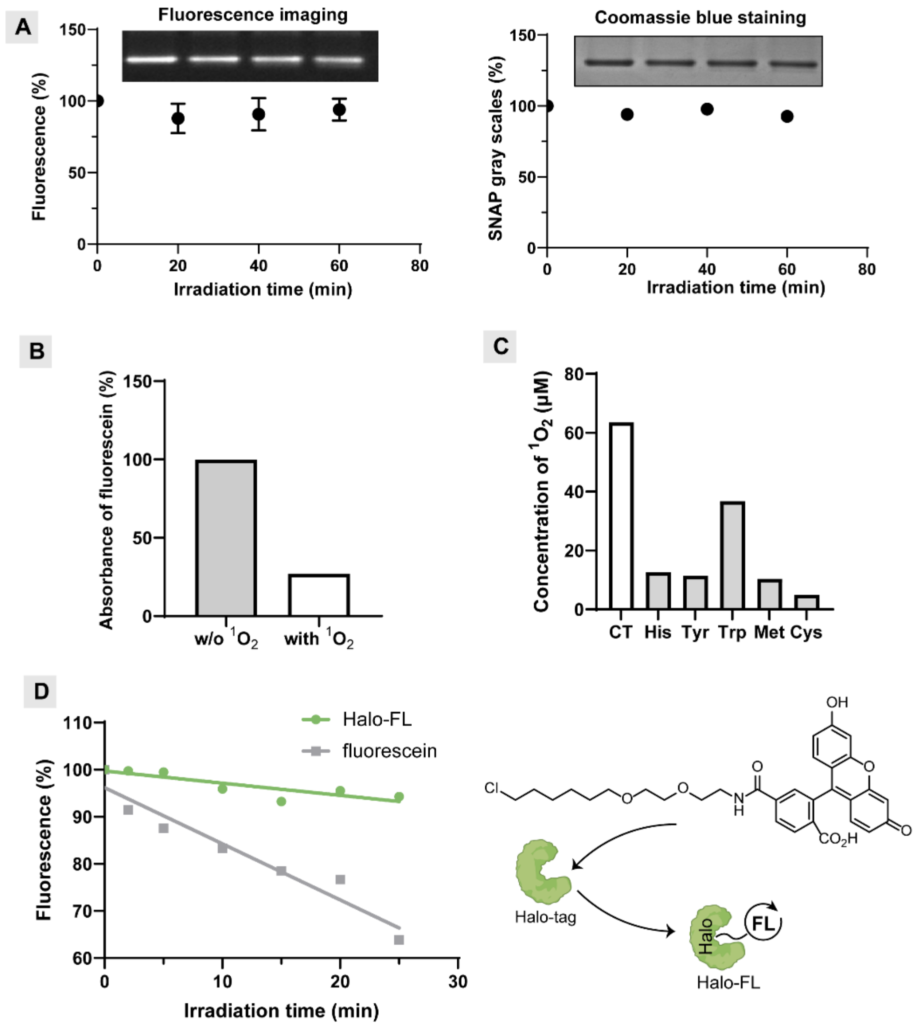

Figure S1. Photostability of SNAP-FL protein. (**A**) In gel analysis of SNAP proteins' (10 μM) stability under 516 nm light irradiation (2.9 mW/cm^2) for 0, 20, 40 and 60 min in the air. (**B**) The oxidative damage of FL (10 μM) by the singlet oxygen ($^1O_2$). The $^1O_2$ was generated by irradiation of methylene blue (50 μM) for 30 s (635 nm, 120 mW/cm^2). (**C**) The $^1O_2$ generated by FL (5 μM) photosensitization (516 nm, 2.9 mW/cm^2, 20 min) and detected by ADPA. The addition of free amino acids (10 mM) quenched the $^1O_2$ in different extents. (**D**) Photostability of Halo-FL protein (5 μM) under 516 nm light irradiation (2.9 mW/cm^2) compared to FL (5 μM).

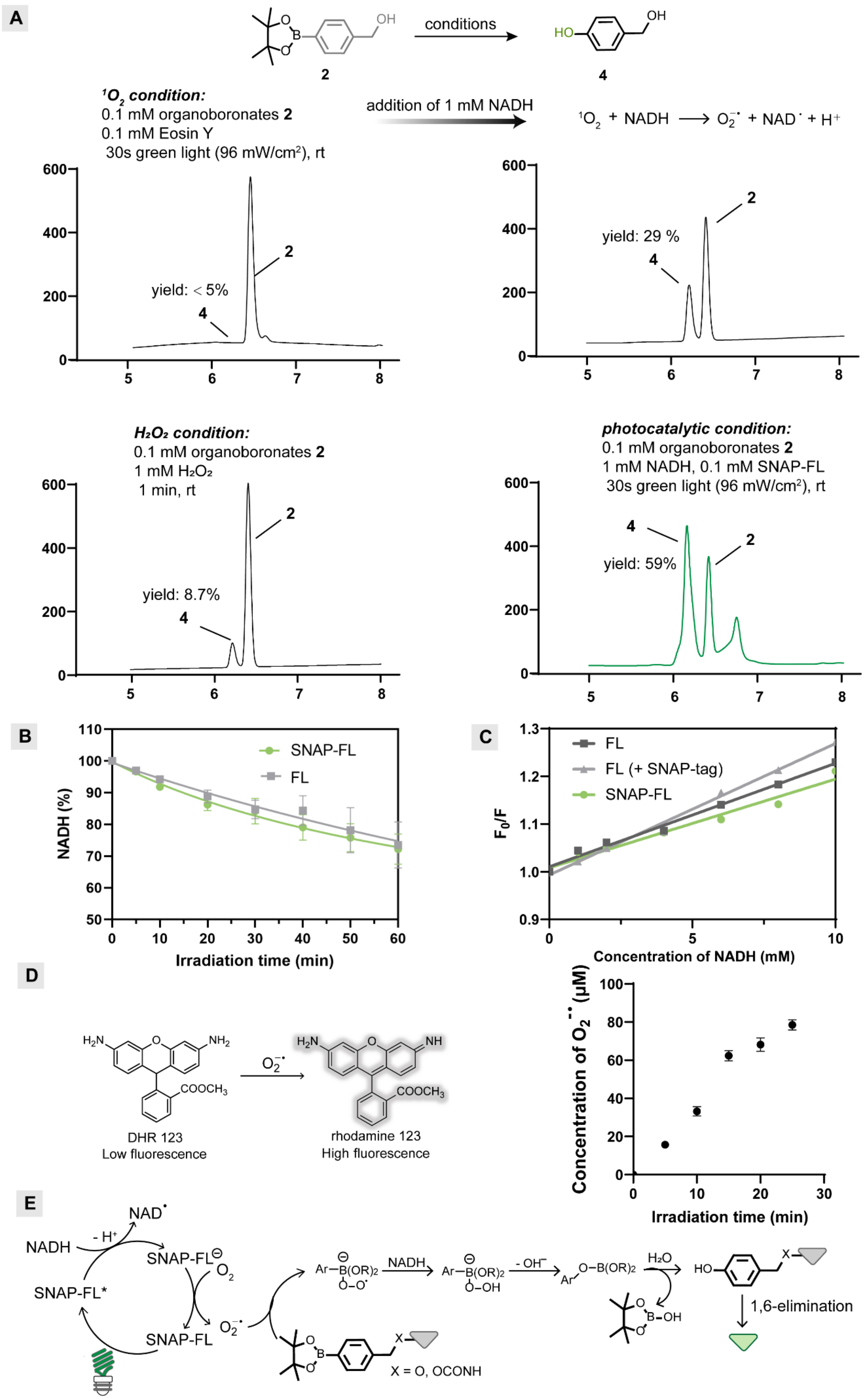
A
conditions
2
4
$^1O_2$ condition:
0.1 mM organoboronates 2
0.1 mM Eosin Y
30s green light (96 mW/cm²), rt
yield: < 5%
addition of 1 mM NADH
$^1O_2$ + NADH ⟶ $O_2^{-\bullet}$ + $NAD^{\bullet}$ + H^+
yield: 29 %
H_2O_2 condition:
0.1 mM organoboronates 2
1 mM H_2O_2
1 min, rt
yield: 8.7%
photocatalytic condition:
0.1 mM organoboronates 2
1 mM NADH, 0.1 mM SNAP-FL
30s green light (96 mW/cm²), rt
yield: 59%
B
SNAP-FL
FL
NADH (%)
Irradiation time (min)
C
FL
FL (+ SNAP-tag)
SNAP-FL
F_0/F
Concentration of NADH (mM)
D
DHR 123
Low fluorescence
rhodamine 123
High fluorescence
Concentration of $O_2^{-\bullet}$ (μM)
Irradiation time (min)
E
NADH
$NAD^{\bullet}$
- H^+
SNAP-FL*
SNAP-FL
O_2
$O_2^{-\bullet}$
- OH^-
H_2O
1,6-elimination
X = O, OCONH

Figure S2. Mechanistic study of photocatalytic deboronative hydroxylation. (**A**) Comparison of the photocatalytic deboronative hydroxylation under indicated conditions with reactive oxygen species. The addition of NADH converts the singlet oxygen to superoxide radical anions for the boronate uncaging. The photocatalytic uncaging is much faster than the hydrogen-peroxide-induced uncaging. (**B**) Kinetic study of NADH consumption by SNAP-FL and FL (1 μM) under 500 nm light irradiation (4.5 mW /cm^2). (**C**) Fluorescence quenching assay to validate the NADH interaction with the excited state of SNAP-FL protein (5 μM). (**D**) Measurement of $O_2^{-\bullet}$ generation from SNAP-FL under 516 nm light irradiation (2.9 mW/cm^2). (**E**) Proposed photocatalytic deboronative hydroxylation mechanism by SNAP-FL.

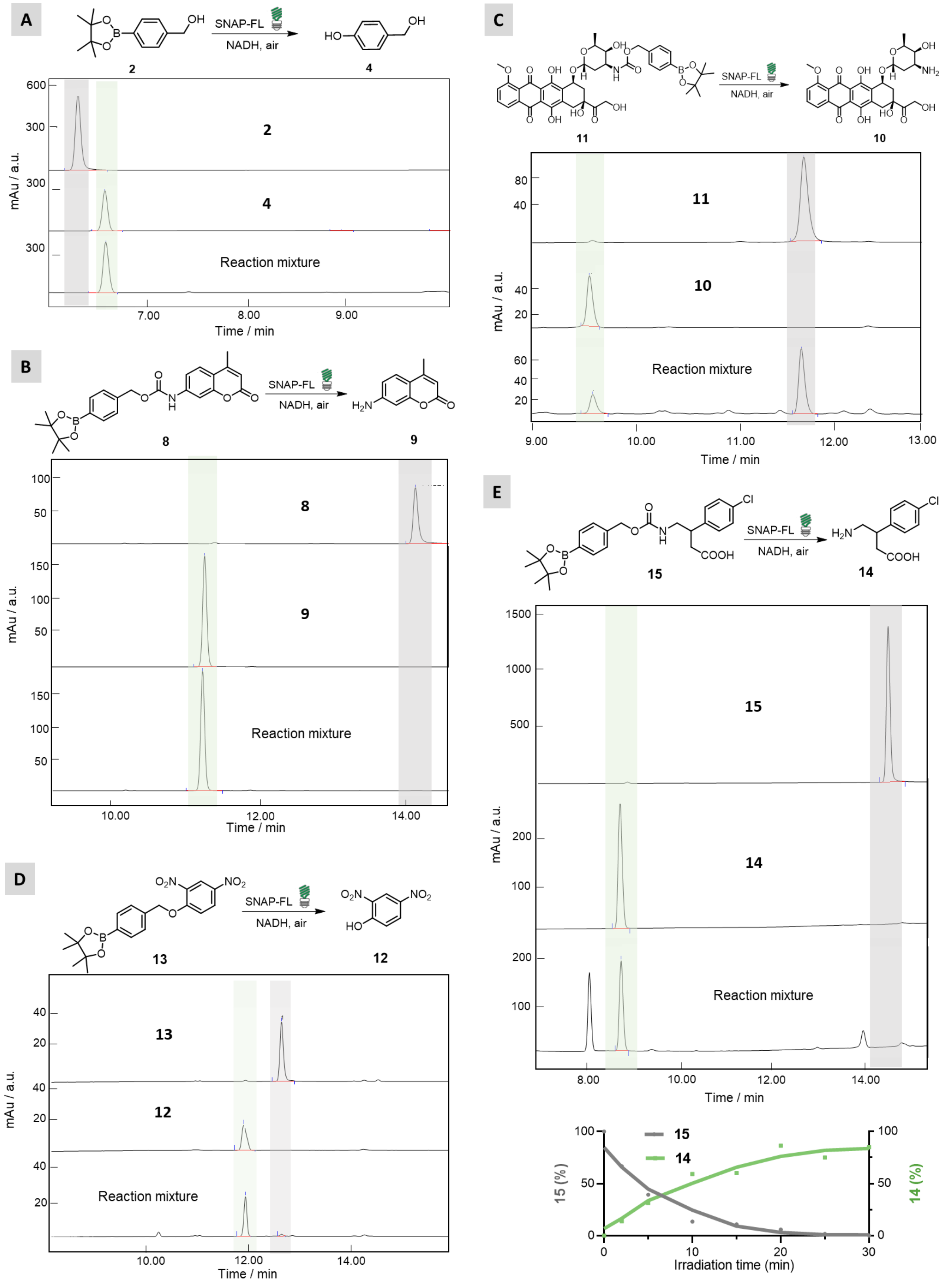

Figure S3. Photocatalytic deboronative hydroxylation of various organoboronates in a cell-free system by HPLC analysis. (**A**) *p*-Hydroxylbenzyl alcohol **4**. (**B**) Aminocoumarin **9**. (**C**) DOX **10**. (**D**) DNP **12**. (**E**) Baclofen **14**.

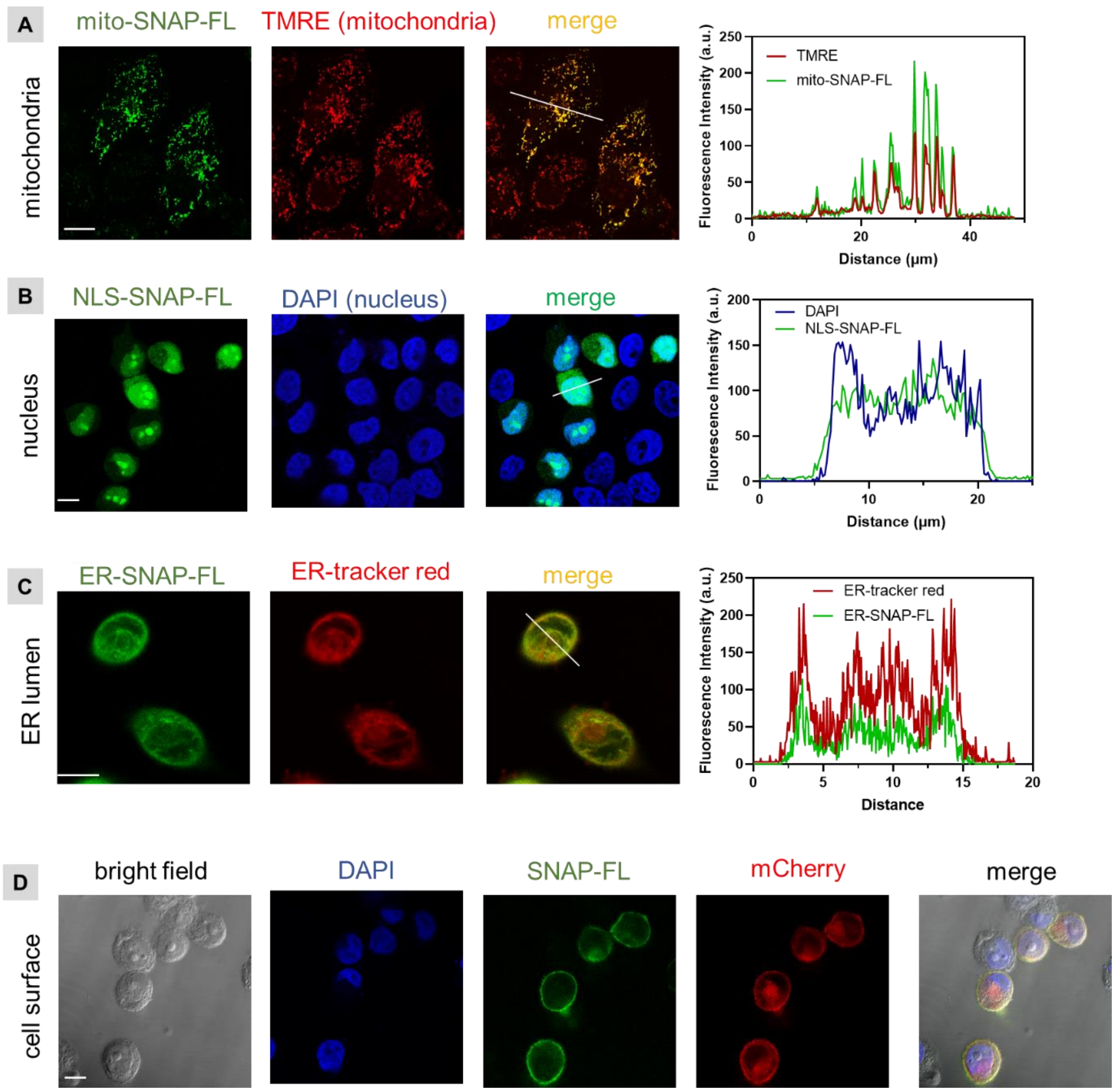

Figure S4. Construction of the subcellular-localized SNAP-FL. Confocal images for SNAP-FL validation targeted mitochondria, nucleus, ER, and the cell surface in HeLa cells. Cells were incubated with CLPDF **7** (2 μM) or BGFL **1** (1 μM) for intracellular and extracellular construction of SNAP-FL, respectively. The color of the DAPI channel was set as blue, the color of FITC channel (SNAP-FL) color was green, and the color of TRITC channel (TMRE, ER tracker and mCherry) was red. Overlays of line profiles show the pixel intensities and the indicated thin white lines. Scale bar: 10 μm.

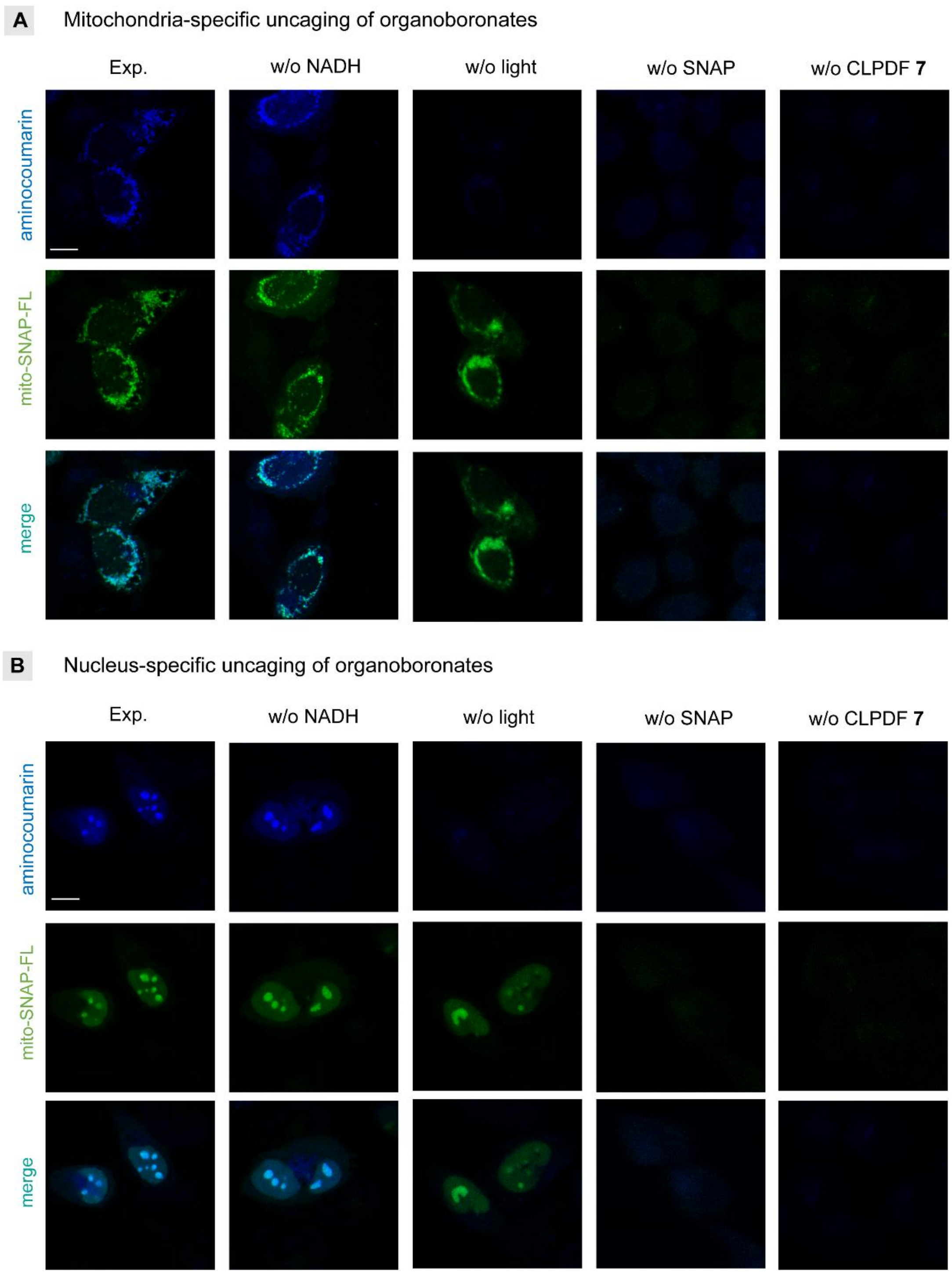

Figure S5. The control experiments of organelle-specific release of aminocoumarin in live cells. (**A**) mito-SNAP-FL releases aminocoumarin **9** with mitochondria-specificity. (**B**) NLS-SNAP-FL releases aminocoumarin **9** with nucleus-specificity. Scale bar: 10 μm.

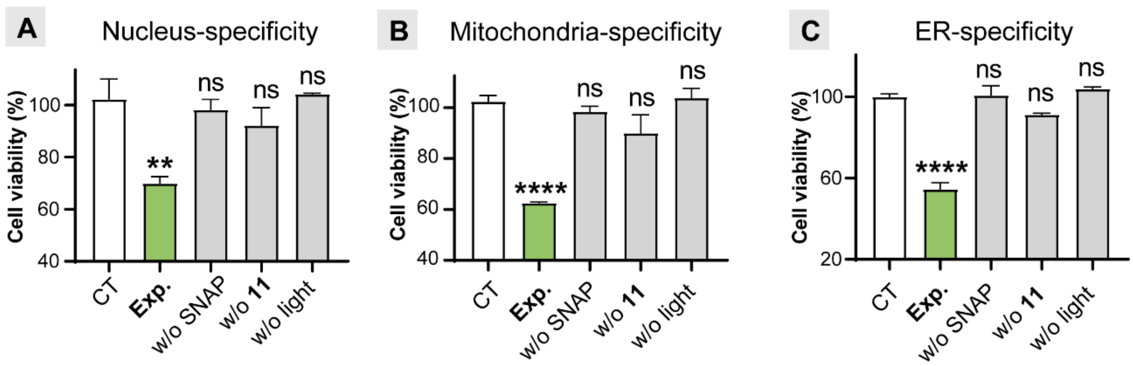

Figure S6. The control experiments of organelle-specific release of DOX. (**A**) NLS-SNAP-FL induced DOX release from organoboronate caged**-**DOX **11**. (**B**) mito-SNAP-FL induced DOX **10** release from organoboronate caged**-**DOX **11**. (**C**) ER-SNAP-FL induced DOX **10** release from organoboronate caged**-**DOX **11**. Exp: experimental group. CT: control group. The statistical significance of differences between groups was evaluated with the unpaired Student's *t* test. ns is not statistically significant. All *p*-values were calculated with control cells treated without transfection, light, or small molecules. A *p*-value of 0.05 and below was considered significant: $p < 0.01$ (**), $p < 0.0001$ (****), ns is not significant. Data are shown as mean ± SEM (n = 3).

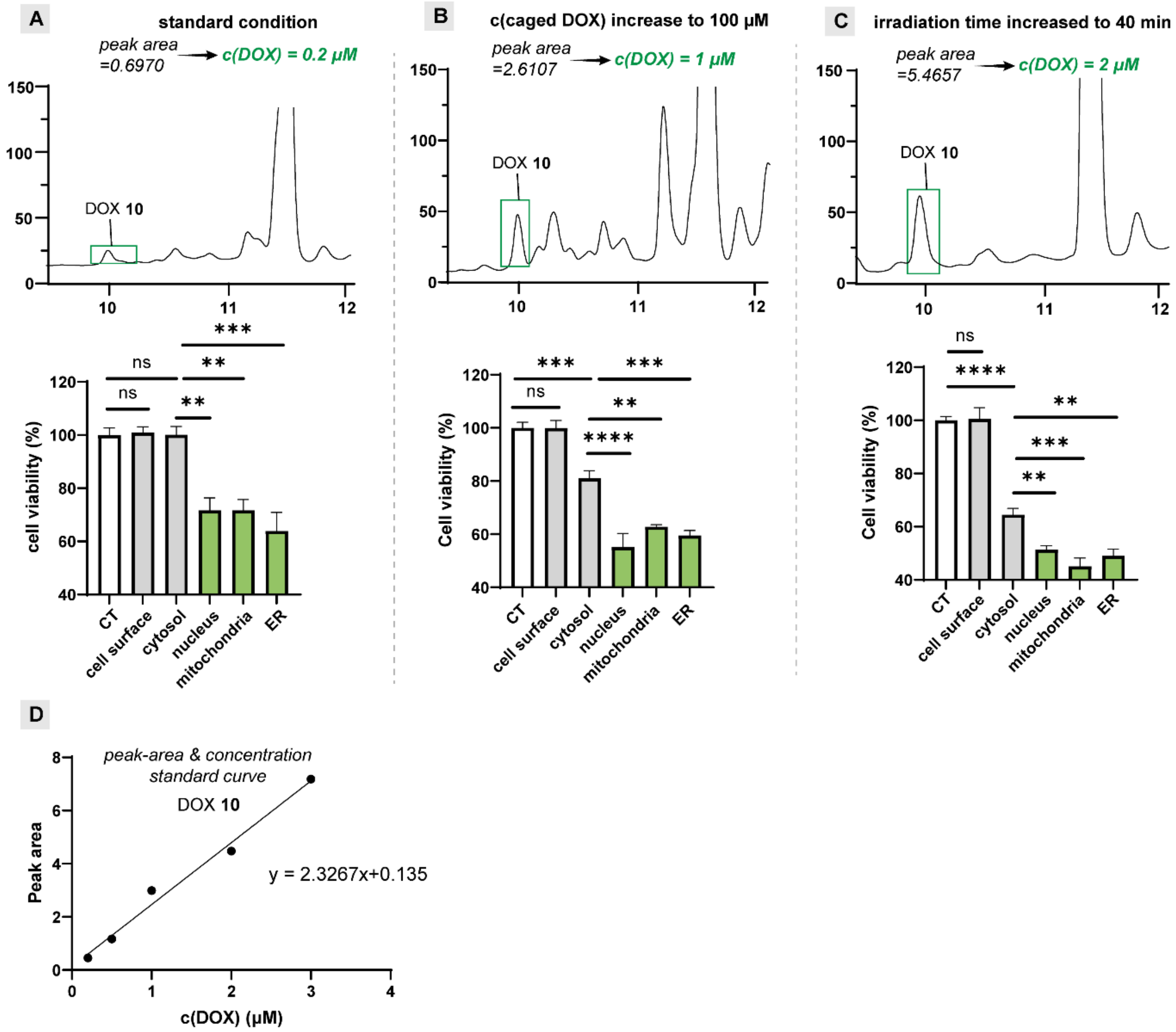

Figure S7. The cell viability from photocatalytic released DOX in different concentrations. (**A**) The estimated concentration of released DOX **10** from peak-area in HPLC analysis (upper) and the cell viability (bottom, as illustrated in Figure 3E) under standard conditions. Standard condition: 20 μM caged DOX **11**, green light irradiation (10 mW/cm^2) for 10 min with 20 μM SNAP-FL. (**B**) The estimated concentration of released DOX from peak-area in HPLC analysis (upper) and the cell viability (bottom) with the elevated dose of caged DOX **11** in 100 μM (n = 5). (**C**) The estimated concentration of released DOX **10** from peak-area in HPLC analysis (upper) and the cell viability (bottom) after the extended light exposure to 40 min (n = 5). (**D**) The standard curve of the peak-area in HPLC analysis with different concentration of DOX **10**. The statistical significance of differences between groups was evaluated with the unpaired Student's t test. A p-value of 0.05 and below was considered significant: $p < 0.01$ (**), $p < 0.001$ (***), $p < 0.0001$ (****), ns is not significant. Data are presented as mean ± SEM (n = 5).

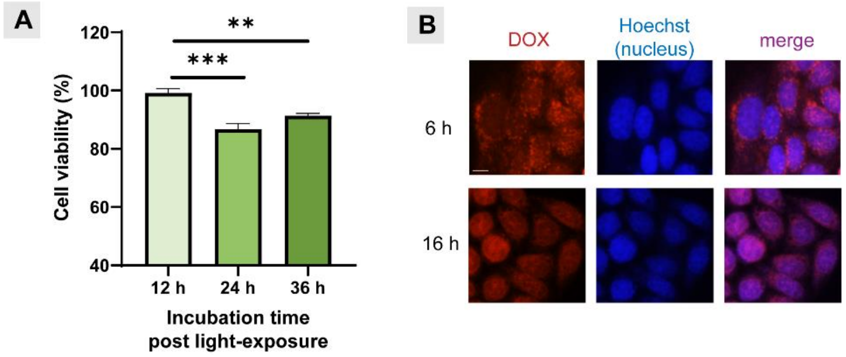

Figure S8. The effect of prolonged incubation time. (**A**) The toxicity from the cytosolic release of DOX **10** with different post-light-exposure incubation (n = 5). Reaction condition: 20 μM caged DOX **11**, green light irradiation (10 mW/cm^2) for 10 min. The statistical significance of differences between groups was evaluated with the unpaired Student's *t* test. A *p*-value of 0.05 and below was considered significant: $p < 0.01$ (**), $p < 0.001$ (***). Data are presented as mean ± SEM. (**B**) The distribution of DOX **10** (0.2 μM) upon different incubation time. The result is similar to previous report.[11] Scale bar: 10 μm.

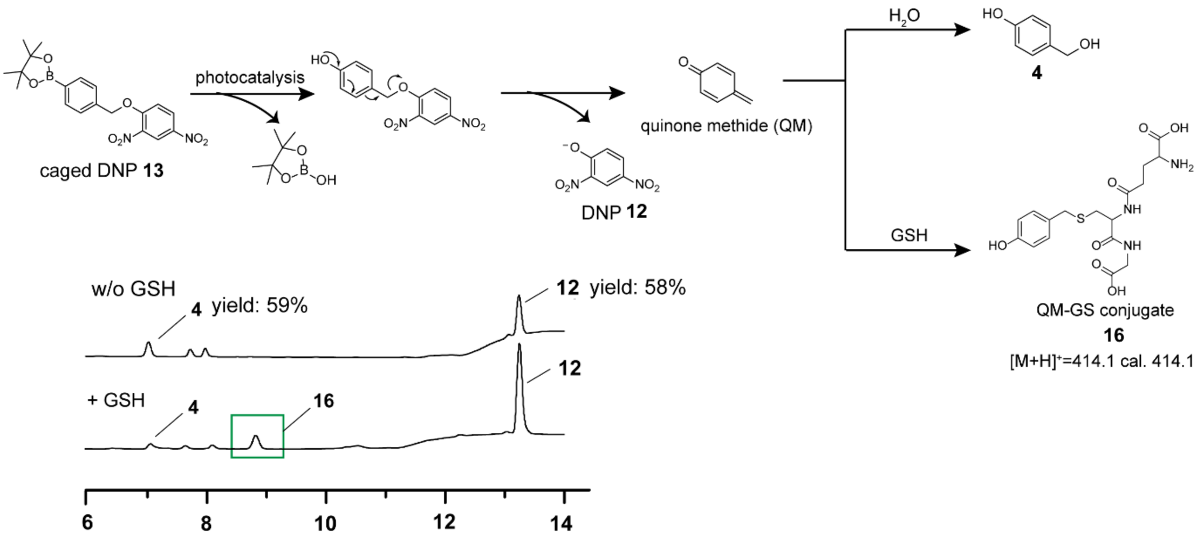

Figure S9. The by-products in photocatalytic uncaging of caged DNP 13. 100 μM of caged DNP **13** was incubated with 10 μM of SNAP-FL protein and 1 mM VcNa under green light irradiation (2.9 mW/cm^2) for 30 min. The concentration of GSH was 10 mM. The reaction mixture was analyzed by HPLC and LC-MS.

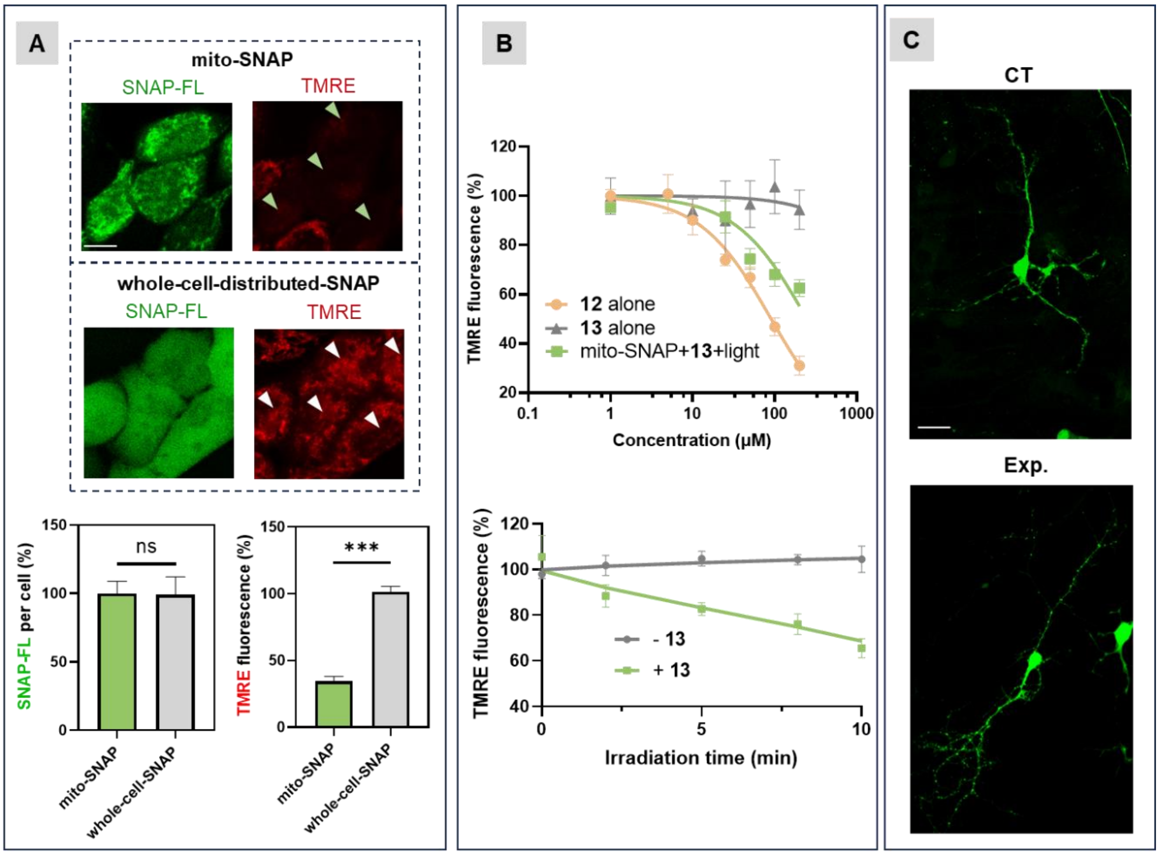

Figure S10. Organelle-specific release of DNP for mitochondrial depolarization. (**A**) Release of DNP by mitochondria-localized SNAP-FL and whole-cell-distributed SNAP-FL. ns, no significant difference, $p < 0.001$ (***), unpaired Student's t test. Data are presented as mean ± SEM. Scale bar: 10 μm. Arrows indicate cells expressing SNAP-FL. (Top) Representative images. (Bottom) Quantification of SNAP-FL and TMRE fluorescence intensities of cells expressing SNAP-FL. (**B**) Dose-/time-dependent of DNP release in mitochondria. (**C**) The fluorescent imaging of group without treatments (CT) and group with optochemical treatment (Exp.) This channel (green) shows mito-SNAP-FL in dendrite and soma of cortical neuron. Scale bar: 20 μm. Data are presented as mean ± SEM.

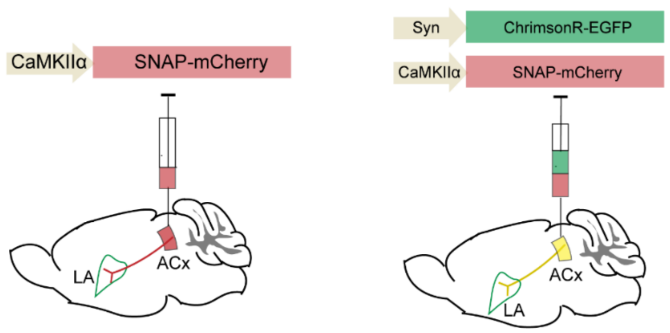

Figure S11. Construction of SNAP-FL in ACx. Schematic AAV injections when photocatalytic uncaging was paired with electrical stimulation (left) or optogenetics (right).

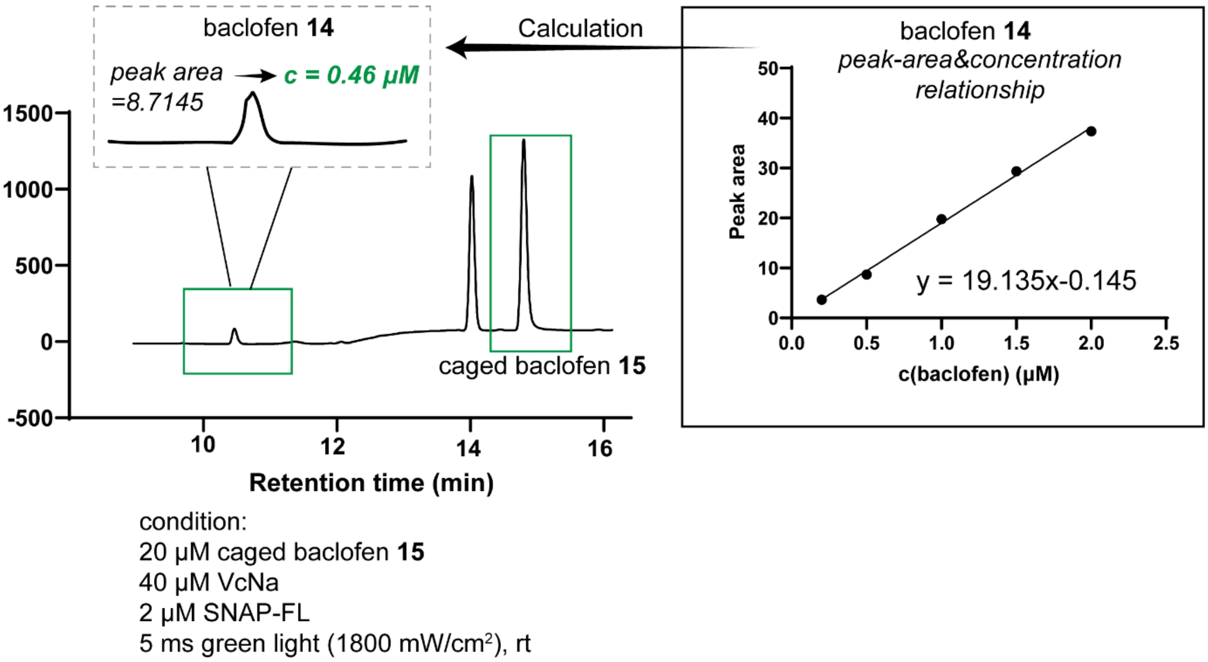

Figure S12. The calculated concentration of released baclofen. 20 µM of caged baclofen **15** was incubated with 2 µM of SNAP-FL and 40 µM VcNa under green light irradiation (1800 mW/cm^2) for 5 ms. The concentration of released baclofen **14** is measured from the peak area in HPLC analysis.

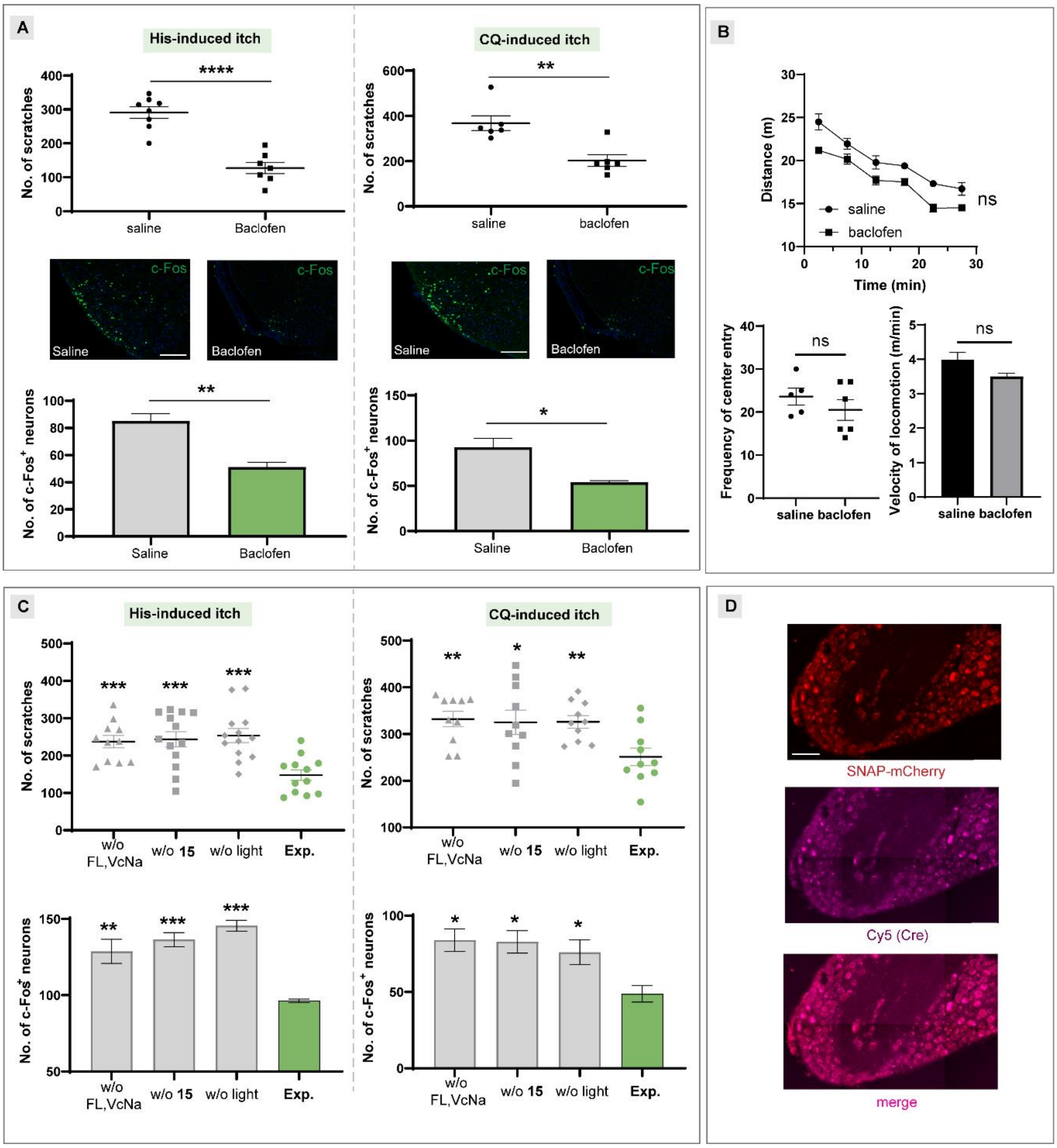

Figure S13. Antipruritic effect of baclofen in live mice. (**A**) Direct injection of baclofen (3.74 mM). Scale bar: 100 μm. (**B**) Baclofen did not affect on locomotor activation and calmness in mice. (**C**) Release of high-dose baclofen from caged baclofen (3.74 mM) by FL (0.9 mM) and VcNa (7.48 mM) under green light irradiation (2.9 mW/cm^2). The antipruritic effect was characterized by the reduced number of scratches (n = 6 or 12) and c-Fos$^+$ neurons (n = 3). (**D**) SNAP-mCherry expression in Na$_V$1.8$^+$ neurons. Scale bar: 100 μm. The statistical significance of the differences between groups was evaluated with the unpaired Student's *t* test. A *p*-value of 0.05 and below was considered significant: $p < 0.05$ (*), $p < 0.01$ (**), $p < 0.001$ (***), ns is not statistically significant. All *p*-values in (**C**) were calculated with the experimental group (Exp.). Data are presented as mean ± SEM.

Table S1. Photocatalytic uncaging of *p*-hydroxylbenzyl alcohol **4**

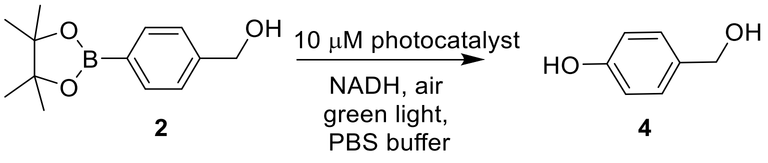

Entry	Conditions	Conversion	Yield
1	SNAP-FL, 1 mM NADH, 30 min	>95%	84%
2	entry 1, sodium ascorbates (VcNa) instead of NADH	57%	43%
3	entry 1, w/o reductant	24%	10%
4	entry 1, dark	<5%	<5%
5	entry 1, w/o photocatalyst	<5%	<5%
6	SNAP-FL, 3 h	>95%	80%
7	FL, 30 min	>95%	88%
8	entry 7, VcNa instead of NADH	>95%	58%

Table S2. Photocatalytic uncaging of aminocoumarin **9**

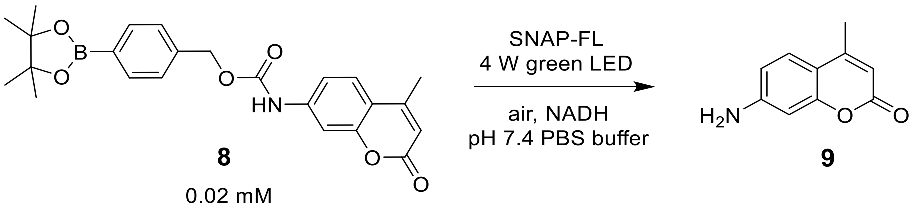

Entry	Conditions	Yield
1	5 μM SNAP-FL, 0.2 mM NADH, 60 min	82%
2	entry 1, dark	<5%
3	entry 1, w/o NADH	5%
4	entry 1, w/o SNAP-FL	<5%

Table S3. Photocatalytic uncaging of DOX **10**

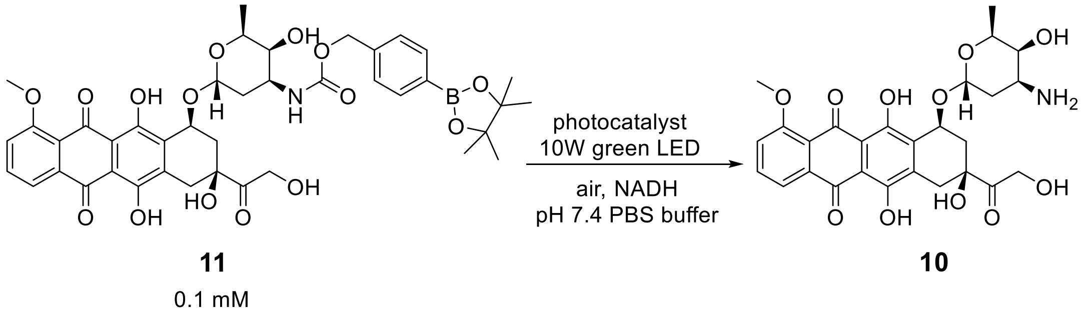

Entry	Conditions	Yield
1	50 μM SNAP-FL, 1 mM NADH, 60 min	18%
2	entry 1, dark	<5%
3	entry 1, w/o NADH	<5%
4	entry 1, w/o SNAP-FL	<5%

Table S4. Photocatalytic uncaging of DNP **12**

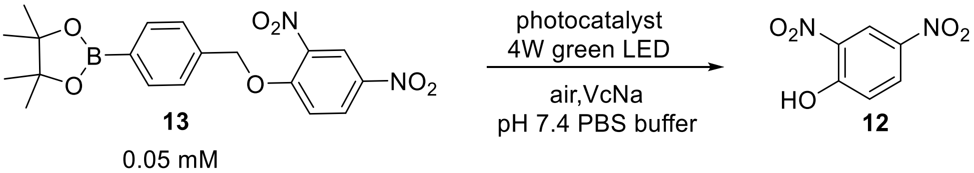

Entry	Conditions	Conversion	Yield
1	10 μM SNAP-FL, 0.5 mM VcNa, 60 min	>95%	66%
2	entry 1, w/o light	<5%	<5%
3	entry 1, w/o SNAP-FL	<5%	<5%

Table S5. Photocatalytic uncaging of baclofen **14**

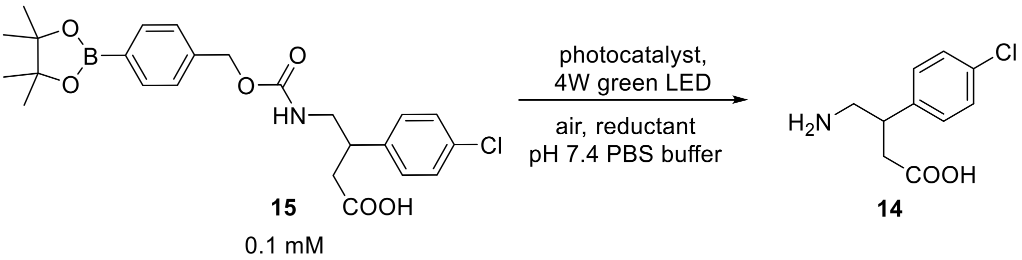

Entry	Conditions	Conversion	Yield
1	10 µM SNAP-FL, 1 mM NADH, 30 min	>95%	73%
2	10 µM FL, 1 mM NADH, 30 min	>95%	85%
3	entry 1, VcNa instead of NADH	69%	46%

3. Genetic Construct

Plasmid

The paragraphs below summarize the plasmids used for peroxidase expression in mammalian and bacterial cells.

SNAP-tag: His-tag (red); SNAP-tag (blue). It was cloned into pSJ2 vector with T7 promoter (for protein expression and purification) and pcDNA 3.1 vector with CMV promoter (for cellular experiments).

MGSHHHHHHHHGSDYDIPTTENLYFQGSMDKDCEMKRTTLDSPLGKLELSG
CEQGLHRIIFLGKGTSAADAVEVPAPAAVLGGPEPLMQATAWLNAYFHQPEAI
EEFPVPALHHPVFQQESFTRQVLWKLLKVVKFGEVISYSHLAALAGNPAATAA
VKTALSGNPVPILIPCHRVVQGDLDVGGYEGGLAVKEWLLAHEGHRLGKPGL
G

Mitochondrial-localized SNAP-tag (mito-SNAP): mitochondrial matrix targeting sequence (red); SNAP-tag (blue). It was cloned into pcDNA 3.1 vector with CMV promoter by Hieff Clone® Plus One Step Cloning Kit (Yeasen).

MLATRVFSLVGKRAISTSVCVRAHGSGSDKDCEMKRTTLDSPLGKLELSGCEQ
GLHRIIFLGKGTSAADAVEVPAPAAVLGGPEPLMQATAWLNAYFHQPEAIEEFP
VPALHHPVFQQESFTRQVLWKLLKVVKFGEVISYSHLAALAGNPAATAAVKT
ALSGNPVPILIPCHRVVQGDLDVGGYEGGLAVKEWLLAHEGHRLGKPGLG

Nucleus-localized SNAP-tag (NLS-SNAP): nuclear targeting sequence (red); SNAP-tag (blue). It was cloned into pcDNA 3.1 vector with CMV promoter.

MDPKKKRKVDPKKKRKVDPKKKRKVGSGSGMDKDCEMKRTTLDSPLGKLE
LSGCEQGLHRIIFLGKGTSAADAVEVPAPAAVLGGPEPLMQATAWLNAYFHQP
EAIEEFPVPALHHPVFQQESFTRQVLWKLLKVVKFGEVISYSHLAALAGNPAA
TAAVKTALSGNPVPILIPCHRVVQGDLDVGGYEGGLAVKEWLLAHEGHRLGK
PGLG

ER-lumen localized SNAP-tag: lg K-chain ss (yellow); HA tag (purple); SNAP-tag (blue); ER-retention sequence (red). It was cloned into pEGFP-C1 vector with CMV promoter, and *egfp* sequence was deleted.

METDTLLLWVLLLWVPGSTGDYPYDVPDYAGSMDKDCEMKRTTLDSPLGK
LELSGCEQGLHRIIFLGKGTSAADAVEVPAPAAVLGGPEPLMQATAWLNAYF
HQPEAIEEFPVPALHHPVFQQESFTRQVLWKLLKVVKFGEVISYSHLAALAGN
PAATAAVKTALSGNPVPILIPCHRVVQGDLDVGGYEGGLAVKEWLLAHEGH
RLGKPGLGGSKDEL

Cytosol-localized SNAP-tag (NES-SNAP): nulear export sequence (red); SNAP-tag (blue). It was cloned into pcDNA 3.1 vector with CMV promoter.

MDKDCEMKRTTLDSPLGKLELSGCEQGLHRIIFLGKGTSAADAVEVPAPAAVL
GGPEPLMQATAWLNAYFHQPEAIEEFPVPALHHPVFQQESFTRQVLWKLLKV
VKFGEVISYSHLAALAGNPAATAAVKTALSGNPVPILIPCHRVVQGDLDVGGY
EGGLAVKEWLLAHEGHRLGKPGLGGSLQLPPLERLTLD

Membrane-tethered SNAP-mCherry: lg K-chain ss (yellow); HA tag (purple); SNAP-tag (blue); PDGFRβ TM domain (orange); mCherry (red). It was cloned into pEGFP-C1 vector with CMV promoter, and *egfp* sequence was deleted.

METDTLLLWVLLLWVPGSTGDYPYDVPDYAGSMDKDCEMKRTTLDSPLGK
LELSGCEQGLHRIIFLGKGTSAADAVEVPAPAAVLGGPEPLMQATAWLNAYF
HQPEAIEEFPVPALHHPVFQQESFTRQVLWKLLKVVKFGEVISYSHLAALAGN
PAATAAVKTALSGNPVPILIPCHRVVQGDLDVGGYEGGLAVKEWLLAHEGH
RLGKPGLGGSGAVGQDTQEVIVVPHSLPFKVVVISAILALVVLTIISLIILIMLW
QKKPRGGSGMVSKGEEDNMAIIKEFMRFKVHMEGSVNGHEFEIEGEGEGRPY
EGTQTAKLKVTKGGPLPFAWDILSPQFMYGSKAYVKHPADIPDYLKLSFPEG
FKWERVMNFEDGGVVTVTQDSSLQDGEFIYKVKLRGTNFPSDGPVMQKKTM
GWEASSERMYPEDGALKGEIKQRLKLKDGGHYDAEVKTTYKAKKPVQLPG
AYNVNIKLDITSHNEDYTIVEQYERAEGRHSTGGMDELYK

Virus

The following viruses were used: AAV-Syn-ChrimsonR-EGFP (Serotype 2/9) and AAV-CaMKIIα-SNAP-mCherry (Serotype 2/9) were produced by Brain VTA (Wuhan, China); AAV-EF1α-DIO-SNAP-mCherry (Serotype 2/PHP.S), AAV-EF1α-DIO-mCherry (Serotype 2/PHP.S), AAV-hSyn-SNAP-mCherry (Serotype 2/PHP.S) and

AAV-hSyn-mCherry (Serotype 2/PHP.S) were packaged by Brain VTA (Wuhan, China). All viral vectors were stored in aliquots at -80 °C until use. The viral titers for injection were more than 10^{12} viral particles per ml.

4. Synthesis of Small Molecules

Synthesis of BGFL **1**

This compound was synthesized according to literature methods.[1-2]

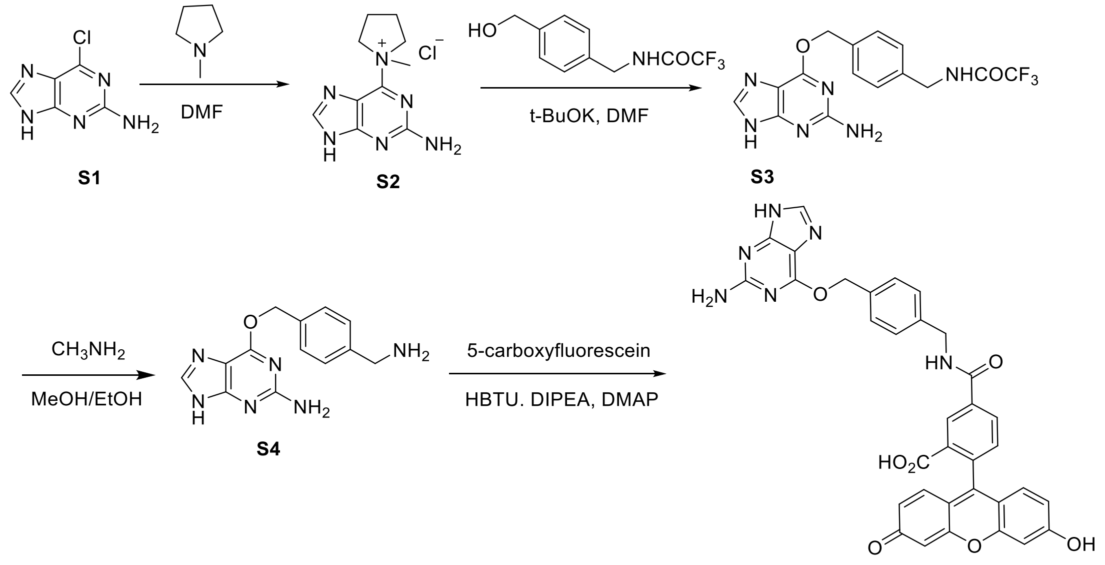

BGFL, **1**

0.80 g (2.4mmol, 1.0 eq.) of 6-chloro-guanine **S1** was dissolved in 16 ml DMF at 50 °C. After cooling to room temperature, 1.2 ml 1-methyl-pyrrolidin (5.7 mmol, 2.0 eq.) was added, and the reaction mixture was stirred overnight. 2.0 ml of acetone was added to complete precipitation at 4 °C. The solid was filtered, washed with ether, and dried in vacuo to give **S2** as white power (0.29 g, 48%). ^{1}H NMR (500 MHz, DMSO-d_6) δ 13.36 (s, 1H), 8.32 (s, 1H), 7.93 (s, 1H), 7.09 (s, 2H), 4.58 (ddt, J = 12.7, 8.2, 4.0 Hz, 2H), 4.04 – 3.86 (m, 2H), 3.63 (s, 3H), 2.29 – 2.17 (m, 2H), 2.09 – 1.97 (m, 2H).^{13}C NMR (126 MHz, DMSO-d_6) δ 159.5, 159.0, 152.1, 143.4, 116.5, 64.5, 52.0, 21.9.

To a solution of 2,2,2-trifluoro-N-(4-hy droxymethyl-benzyl)-acetamide (2.8 g, 12 mmol，3.0 eq.) in 50 mL DMF, potassium-t-butoxide (2.7 g, 24 mmol，6.0 eq.) was added. 1-(2-Amino-7H-purin6-yl)-1-methyl-pyrrolidinium chloride **S2** (1. 0 g, 4.0 mmol，1.0 eq.) was then added, and the solution was stirred overnight. The residue was purified by silica gel column chromatography (methanol/dichloromethane 1/10) to

give **S3** (1.3 g, 85 %) as a yellow solid. ^{1}H NMR (500 MHz, DMSO-d_6) δ 12.39 (s, 1H), 9.99 (t, *J* = 5.9 Hz, 1H), 7.78 (s, 1H), 7.54 – 7.41 (m, 2H), 7.38 – 7.20 (m, 2H), 6.27 (s, 2H), 5.44 (s, 2H), 4.38 (d, *J* = 5.9 Hz, 2H). ^{13}C NMR (126 MHz, $CDCl_3$) δ 165.0, 164.8, 161.7, 161.4, 160.4, 142.4, 141.1, 133.9, 132.7, 122.4, 120.1, 71.6, 47.6.

To a solution of N-[4-(2-amino-9H-purin-6-yloxymethyl)-benzyl]-2,2,2-trifluoro-acetamide **S3** (1.0 g, 2.7 mmol，1.0 eq.) in 15 mL methanol, methylamine (33% in ethanol, 15.0 mL) was added. The mixture was stirred at room temperature for 24 h. The solvent was removed in vacuo, providing product **S4** as a white powder (0.60 g, 82% yield). ^{1}H NMR (500 MHz, DMSO-d_6) δ 7.80 (s, 1H), 7.42 (d, *J* = 8.0 Hz, 2H), 7.33 (d, *J* = 7.9 Hz, 2H), 6.25 (s, 2H), 5.43 (s, 2H), 3.71 (s, 2H). ^{13}C NMR (126 MHz, DMSO-d_6) δ 160.0, 159.9, 156.4, 141.9, 138.9, 135.6, 128.9, 128.0, 126.8, 67.0, 45.0.

To a solution of O^6-(4-aminomethyl-benzyl) guanine **S4** (0.11 g, 0.40 mmol, 1.0 eq.), 5-carboxylfluorescein (5-FAM, 0.15 g, 0.40 mmol, 1.0 eq.), 4-dimethylaminopyridine (DMAP, 0.016g，0.12 mmol, 0.30 eq.) and O-benzotriazole-N,N,N',N'-tetramethyl-uronium-hexafluorophosphate (HBTU, 0.16 g, 0.48 mmol, 1.2 eq.) in 5.0 mL DMF, *N,N*-diisopropylethylamine (DIPEA, 0.20 mL, 1.2 mmol, 3.0 eq.) was added dropwise. The reaction was stirred at room temperature overnight, then concentrated in a vacuum. The residue was purified by silica gel column chromatography (methanol/dichloromethane 1/10) to give BGFL **1** (0.12 g, 48%) as a red solid. ^{1}H NMR (500 MHz, DMSO-d_6) δ 12.39 (s, 1H), 10.18 (s, 1H), 10.15 (s, 1H), 9.39 (s, 1H), 8.49 (s, 1H), 8.27 (d, *J* = 8.0 Hz, 1H), 7.78 (s, 1H), 7.47 (d, *J* = 8.2 Hz, 2H), 7.36 (d, *J* = 7.0 Hz, 2H), 7.26 (d, *J* = 9.0 Hz, 1H), 6.67 (s, 2H), 6.65 – 6.40 (m, 4H), 6.26 (s, 2H), 5.45 (s, 2H), 4.52 (s, 2H). ^{13}C NMR (151 MHz, DMSO-d_6) δ 168.6, 165.1, 160.3, 160.1, 155.2, 152.3, 142.7, 139.6, 138.3, 136.5, 135.9, 135.2, 129.7, 129.1, 127.9, 127.0, 124.8, 123.9, 113.1, 109.5, 107.3, 102.7, 83.8, 67.0, 43.2. IR (neat): 3427, 1741, 1624, 1465, 1248, 1210, 1180, 1112, 1033, 1015, 668 cm^{-1}. HRMS-ESI (m/z) [$M+H^+$] calc'd. for $C_{34}H_{25}N_6O_7$ 629.1779, found 629.1778.

<u>Synthesis of CLPDF **7**</u>

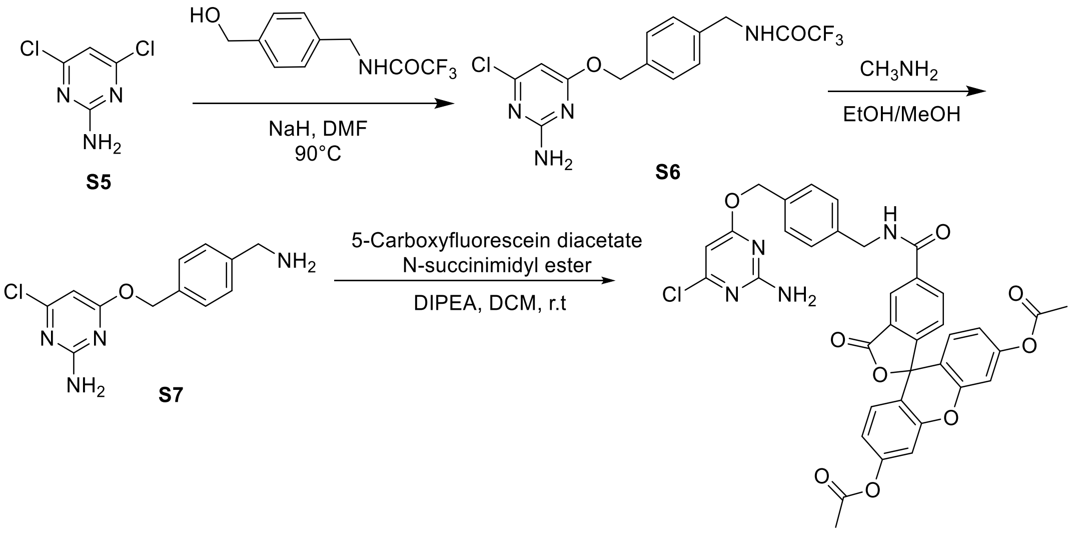

CLPDF, **7**

To a solution of 2,2,2-trifluoro-N-(4-hy droxymethyl-benzyl)-acetamide (0.24 g, 1.5 mmol，1.0 eq.) in 5 mL DMF, NaH (60% in mineral oil, 0.18 g, 4.4 mmol) was added slowly. After stirring at room temperature for 15 min, a solution of 4,6-dichloropyrimidin-4-amine **S5** (0.34 mg, 1.5 mmol, 1.0 eq.) in anhydrous DMF (5 mL) was added dropwise to the mixture. The reaction was then heated to 90 °C for 6 h. The reaction was cooled to room temperature, quenched with a slow addition of cold water (10 mL), extracted with ethyl acetate (3 × 20 mL), and dried over Na_2SO_4. The residue was purified by silica gel column chromatography (ethyl acetate: petroleum ether 2/1) to give **S6** as a white solid (0.12g, 22% yield). ^{1}H NMR (500 MHz, DMSO-d_6) δ 9.99 (t, J = 5.9 Hz, 1H), 7.40 (d, J = 8.1 Hz, 2H), 7.27 (d, J = 8.0 Hz, 2H), 7.09 (s, 2H), 6.12 (s, 1H), 5.28 (s, 2H), 4.37 (d, J = 6.0 Hz, 2H). ^{13}C NMR (126 MHz, CD_3OD) δ 170.9, 162.6, 160.3, 136.9, 135.6, 128.3, 127.7, 96.1, 67.7, 42.9.

To a solution of compound **S6** (86 mg, 0.24 mmol，1.0 eq.) in 3.0 mL methanol, methylamine (33% in EtOH, 1.0 mL) was added. The solvent was removed in vacuo, providing product **S7** as a white powder (55 mg, 95% yield). ^{1}H NMR (500 MHz, CD_3OD) δ 7.47 – 7.35 (m, 4H), 6.09 (s, 1H), 5.35 (s, 2H), 3.91 (s, 2H). ^{13}C NMR (126 MHz, CD_3OD) δ 170.9, 160.4, 138.4, 136.1, 128.3, 127.8, 95.1, 67.4, 44.0.

To a solution of 5-carboxylfluorescein diacetate N-succinimidyl ester (17 mg, 0.03 mmol, 1.0 eq.) in 2 mL DCM, compound **S7** (10 mg, 0.0375 mmol, 1.25 eq.) was added. The reaction was then stirred at room temperature for 2 h, and the residue was purified

by column chromatography on silica gel, giving CLPDF **7** as a white solid (6 mg, 26% yield). ^{1}H NMR (500 MHz, $CDCl_3$) δ 8.44 (s, 1H), 8.23 (dd, *J* = 8.0, 1.5 Hz, 1H), 7.34 (q, *J* = 7.9 Hz, 4H), 7.24 (d, *J* = 8.0 Hz, 1H), 7.10 (d, *J* = 2.2 Hz, 2H), 6.84 – 6.72 (m, 4H), 6.13 (s, 1H), 5.27 (s, 2H), 5.22 (s, 2H), 4.65 (d, *J* = 5.6 Hz, 2H), 2.31 (s, 6H). ^{13}C NMR (126 MHz, $CDCl_3$) δ 170.8, 168.9, 168.4, 165.4, 162.2, 160.9, 155.2, 152.3, 151.5, 137.9, 136.7, 135.5, 135.1, 128.8, 128.5, 128.2, 126.5, 124.6, 123.3, 118.0, 115.6, 110.6, 97.2, 82.2, 67.9, 44.0, 21.1. IR (neat): 1760, 1611, 1552, 1422, 1284, 1270, 1246, 1203, 1155, 1111, 1038, 1027, 759, 734 cm^{-1}. HRMS-ESI (m/z) [M+H$^+$] calc'd. for $C_{37}H_{28}ClN_4O_9$ 707.1539, found 707.1541.

<u>Synthesis of caged aminocoumarin **8**</u>

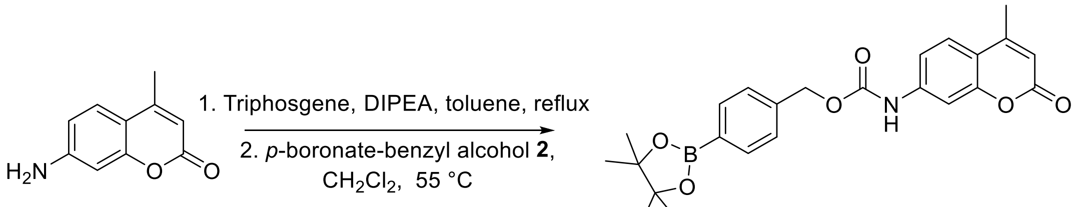

9 **8**

Triphosgene (0. 14 g, 0.46 mmol, dissolved in 4 mL toluene) was slowly added to a solution of 7-amino-4-methylcoumarin **8** (69 mg, 0.39 mmol, 1.0 eq.), and DIPEA (250 mL, 1.45 mmol, 3.8 eq.) in 4 mL toluene at 0 °C. The reaction mixture was refluxed with stirring under a nitrogen atmosphere for 4 h, and then cooled to ambient temperature. CH_2Cl_2 (3.0 mL) was added and stirred for 5 min until the solution turned dark brown, and *p*-boronate ester-benzyl alcohol **2** (0. 12 mg, 0.5 mmol, 1.3 eq., in 5 mL CH_2Cl_2) was added. The reaction mixture was stirred, heated at 55 °C for 13 h, and then cooled to ambient temperature. The resulting solution was concentrated to dryness under reduced pressure. The residue was purified by column chromatography on silica gel (methanol/dichloromethane 1/25) to give caged aminocoumarin **9** as a white solid (11 mg, 84% yield). ^{1}H NMR (500 MHz, $CDCl_3$) δ 7.82 (d, *J* = 7.9 Hz, 2H), 7.51 (d, *J* = 8.6 Hz, 1H), 7.44 (d, *J* = 1.7 Hz, 1H), 7.40 (d, *J* = 7.7 Hz, 2H), 7.03 (s, 1H), 6.18 (s, 1H), 5.24 (s, 2H), 2.40 (s, 3H), 1.34 (s, 12H). ^{13}C NMR (126 MHz, $CDCl_3$) δ 161.1, 154.4, 152.7, 152.2, 141.3, 138.5, 135.1, 127.4, 125.4, 115.6, 114.4, 113.2, 105.9, 102.0, 83.9, 67.3, 29.7, 24.8, 18.6. IR (neat): 3270, 2978, 1728, 1698, 1690, 1615, 1587, 1558,

1533, 1423, 1398, 1361, 1318, 1269, 1239, 1145, 1087, 1065, 963, 860, 818, 750, 656 cm^{-1}. HRMS-ESI (m/z) [$M+H^+$] calc'd. for $C_{24}H_{27}BNO_6$ 435.1962, found 435.1964.

Synthesis of caged DOX **11**

This compound was synthesized according to the literature methods.[12]

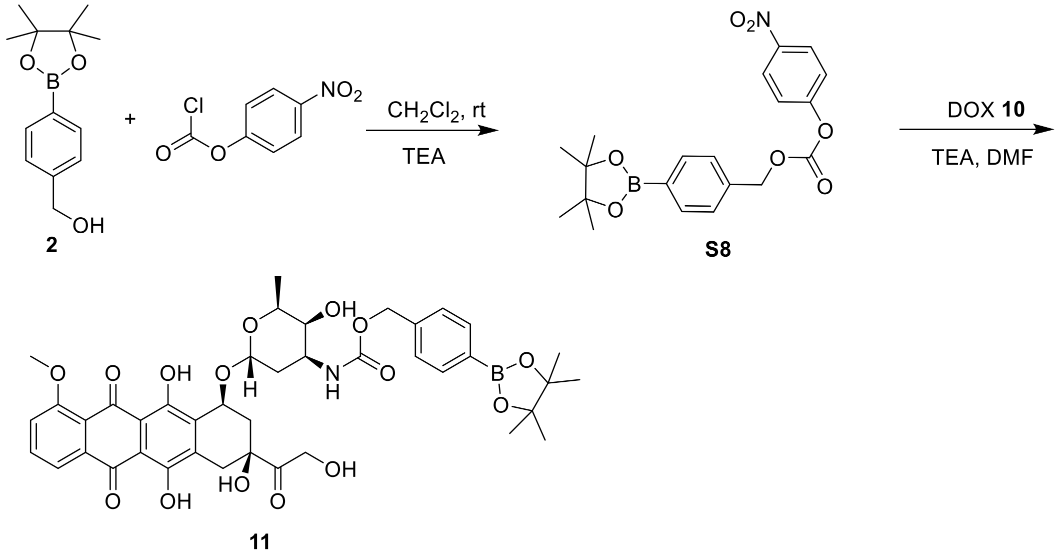

p-boronate ester-benzyl alcohol **2** (0.47 g, 2.0 mmol, 1.0 eq.) was dissolved in 5 mL tetrahydrofuran. Then, *p*-nitrobenzoyl chloride (0.51 g, 2.5 mmol, 1.25 eq.) and triethylamine (TEA, 0.6 mL, 4.0 mmol, 2.0 eq.) were added. The mixture was stirred at room temperature overnight. The resulting solution was concentrated to dryness under reduced pressure. The residue was purified by column chromatography on silica gel (ethyl acetate: petroleum ether 1/16) to give compound **S8** as a white solid (0.36 g, 47% yield). ^1H NMR (500 MHz, $CDCl_3$) δ 8.27 (d, J = 9.0 Hz, 2H), 7.85 (d, J =8.0 Hz, 2H), 7.44 (d, J = 7.5 Hz, 2H), 7.38 (d, J = 9.0 Hz, 2H), 5.30 (s, J = 6.0 Hz, 2H), 1.35 (s, 12H).

Doxorubicin hydrochloride **10** (DOX, 22 mg, 0.04 mmol, 1.0 eq.), compound **S8** (32 mg, 0.08 mmol, 2.0 eq.), and TEA (17 µL, 0.12 mmol, 3.0 eq.) were dissolved in 1.0 mL of anhydrous DMF and reacted at room temperature in the dark for overnight. then diluted with ethyl acetate 10 mL and washed with water (10 mL×3 times). The combined organic layer was washed with brine, dried over Na_2SO_4, and concentrated in a vacuum. The residue was purified by column chromatography on silica gel

(methanol/dichloromethane 1/16) to give caged DOX **11** as a red solid (11 mg, 33%). ^{1}H NMR (500 MHz, $CDCl_3$) δ 8.04 (d, *J* = 7.6 Hz, 1H), 7.79 (d, *J* = 8.1 Hz, 1H), 7.78 – 7.72 (m, 2H), 7.39 (d, *J* = 8.5 Hz, 1H), 7.30 (d, *J* = 7.6 Hz, 2H), 5.50 (d, *J* = 3.9 Hz, 1H), 5.34 – 5.26 (m, 2H), 5.04 (s, 2H), 4.75 (d, *J* = 3.8 Hz, 2H), 4.58 (s, 1H), 4.14 (d, *J* = 6.7 Hz, 1H), 4.08 (s, 3H), 3.87 (s, 1H), 3.66 (s, 1H), 3.27 (s, 1H), 3.02 (d, *J* = 5.5 Hz, 1H), 2.18 (d, *J* = 3.9 Hz, 1H), 1.99 (m, 2H), 1.32 (s, 12H), 1.28 (d, *J* = 6.6 Hz, 3H). HRMS-ESI (m/z) [M+Na$^+$] calc'd. for $C_{41}H_{46}BNNaO_{15}$ 826.2858 found 826.2867.

Synthesis of caged DNP **13**

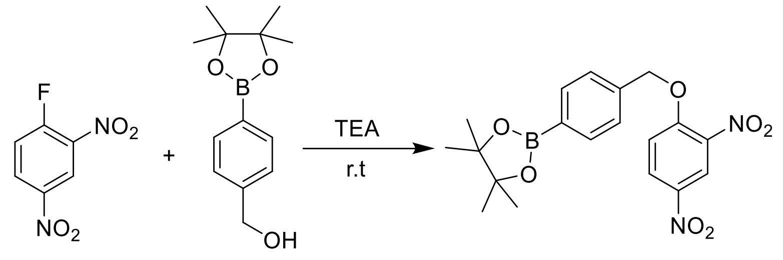

2 **13**

p-boronate ester-benzyl alcohol **2** (0.375 g, 1.60 mmol, 1.0 eq.) was mixed with 2,4-dinitrofluorobenzene (1.17 mL, 10.0 mmol, 6.3 eq.) and ten drops of anhydrous triethylamine was added. The reaction mixture was stirred at room temperature overnight. The resulting solution was concentrated to dryness under reduced pressure. The residue was purified by column chromatography on silica gel (petroleum ether/ethyl acetate = 5/1) to give caged DNP **13** as a white solid (0.220 g, 34% yield). ^{1}H NMR (500 MHz, $CDCl_3$) ^{1}H NMR (500 MHz, $CDCl_3$) δ 8.8 (d, J = 2.8 Hz, 1H), 8.4 (dd, J = 9.3, 2.8 Hz, 1H), 7.8 (d, J = 8.0 Hz, 2H), 7.4 (d, J = 8.0 Hz, 2H), 7.2 (d, J = 9.3 Hz, 1H), 5.4 (s, 2H), 1.3 (s, 12H).^{13}C NMR (151 MHz, $CDCl_3$) 156.2, 140.3, 139.3, 136.9, 135.4, 128.9, 126.2, 121.9, 115.0, 84.0, 72.0, 24.9. IR (neat): 2979, 1536, 1490, 1287, 1144, 1089, 1070, 1015, 963, 922, 858, 832, 743, 656 cm^{-1}. HRMS-ESI (m/z) [M+Na$^+$] calc'd. for $C_{19}H_{21}BN_2NaO_7$ 422.1370 found 422.1377.

5. NMR Spectra of New Compounds

1: ^{1}H-NMR (500 MHz, $CDCl_3$)

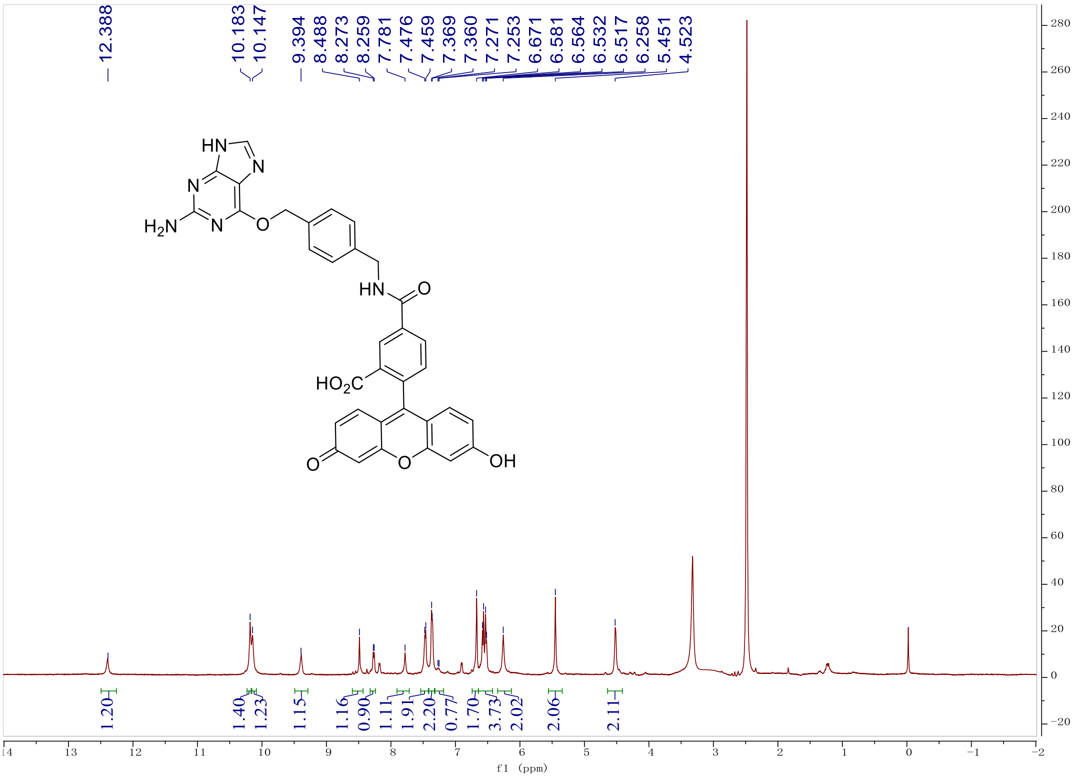

1: ^{13}C-NMR (151MHz, $CDCl_3$)

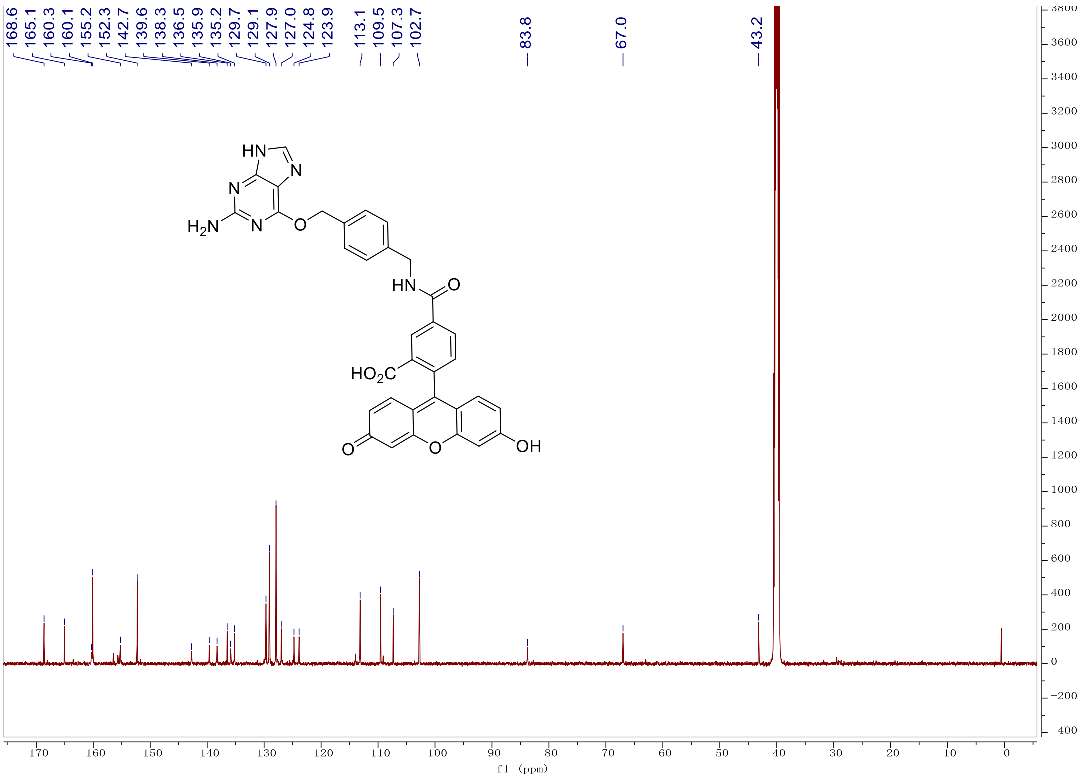

7: ^{1}H-NMR (500 MHz, $CDCl_3$)

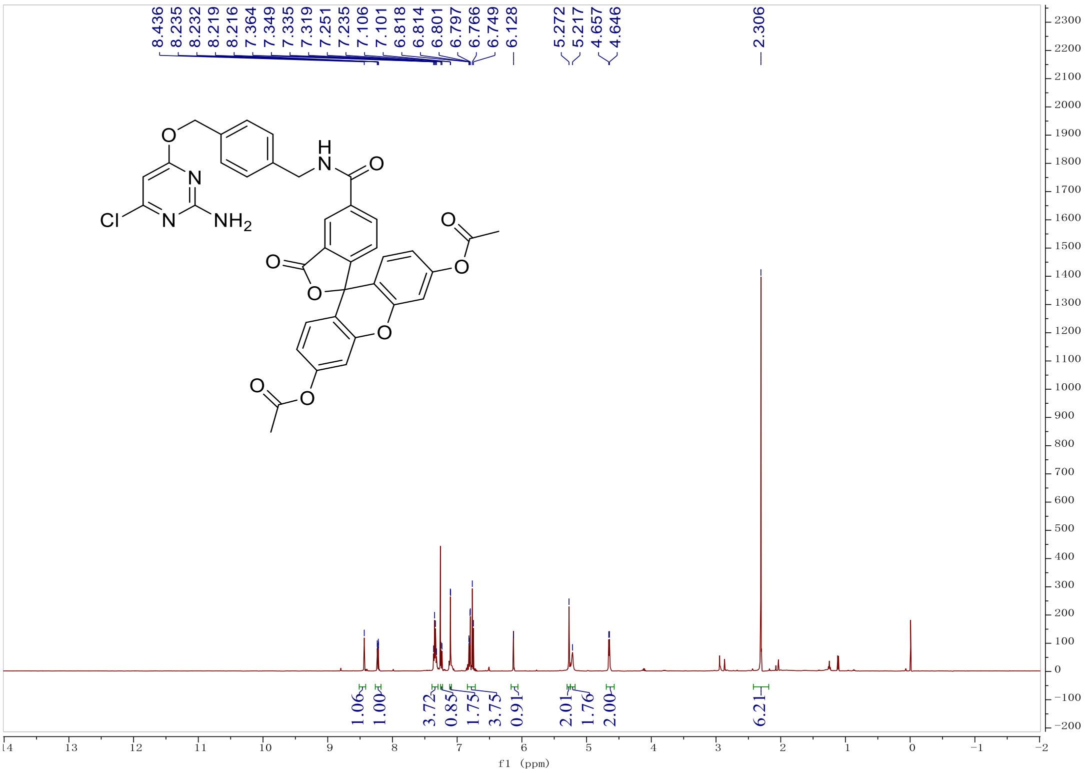

7: ^{13}C-NMR (126MHz, $CDCl_3$)

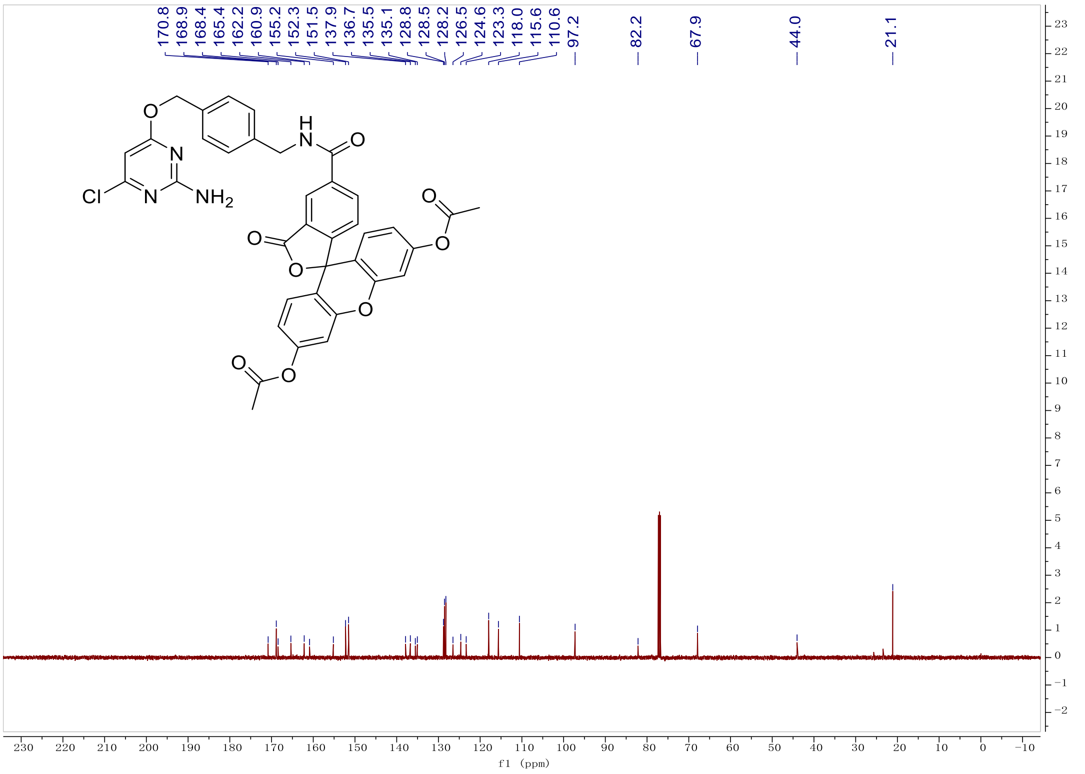

8: ^{1}H-NMR (500 MHz, $CDCl_3$)

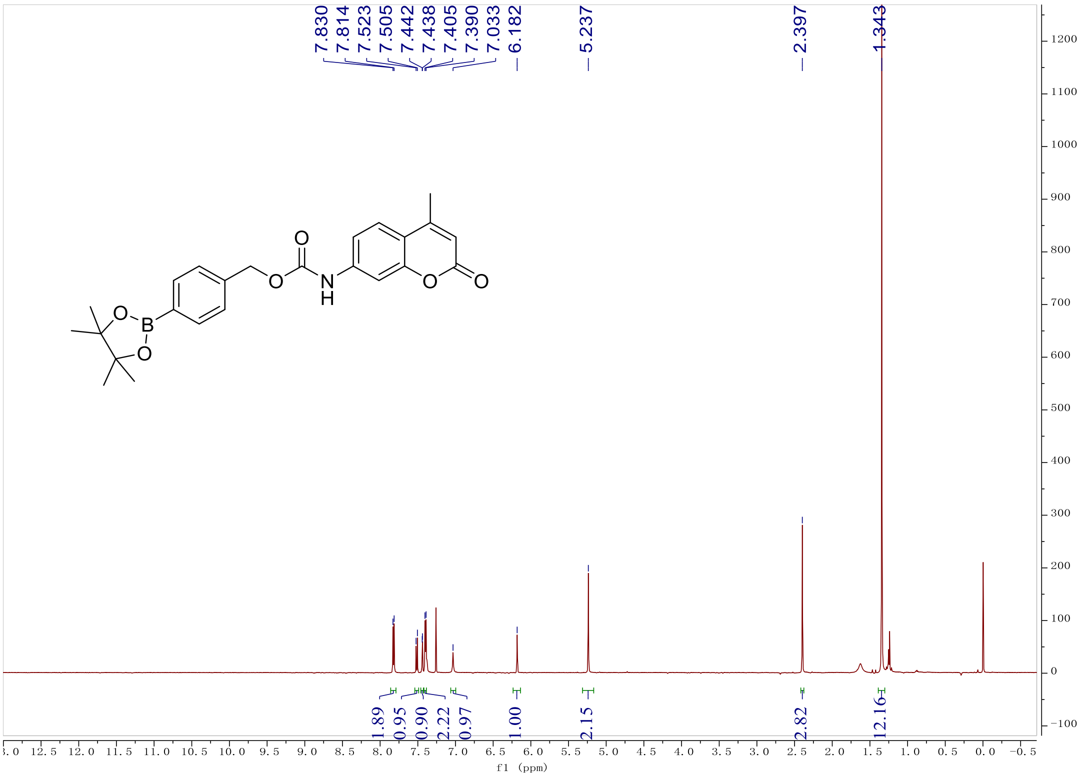

8: ^{13}C-NMR (126 MHz, $CDCl_3$)

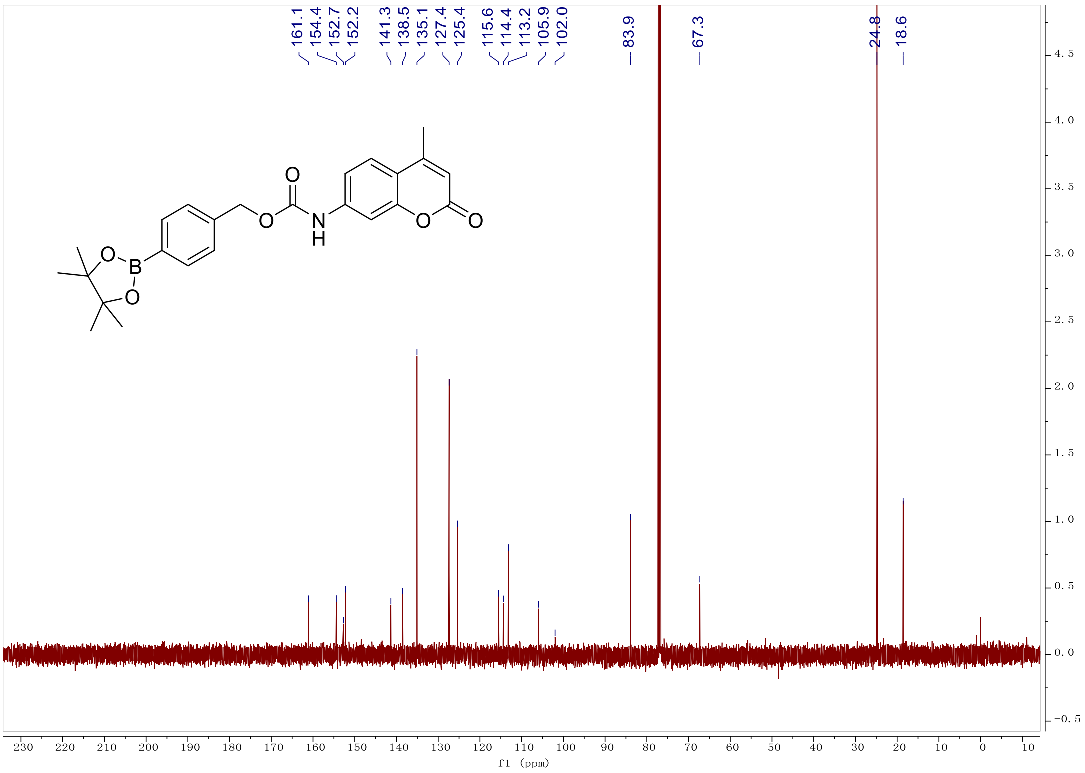

13: ^{1}H-NMR (500 MHz, $CDCl_3$)

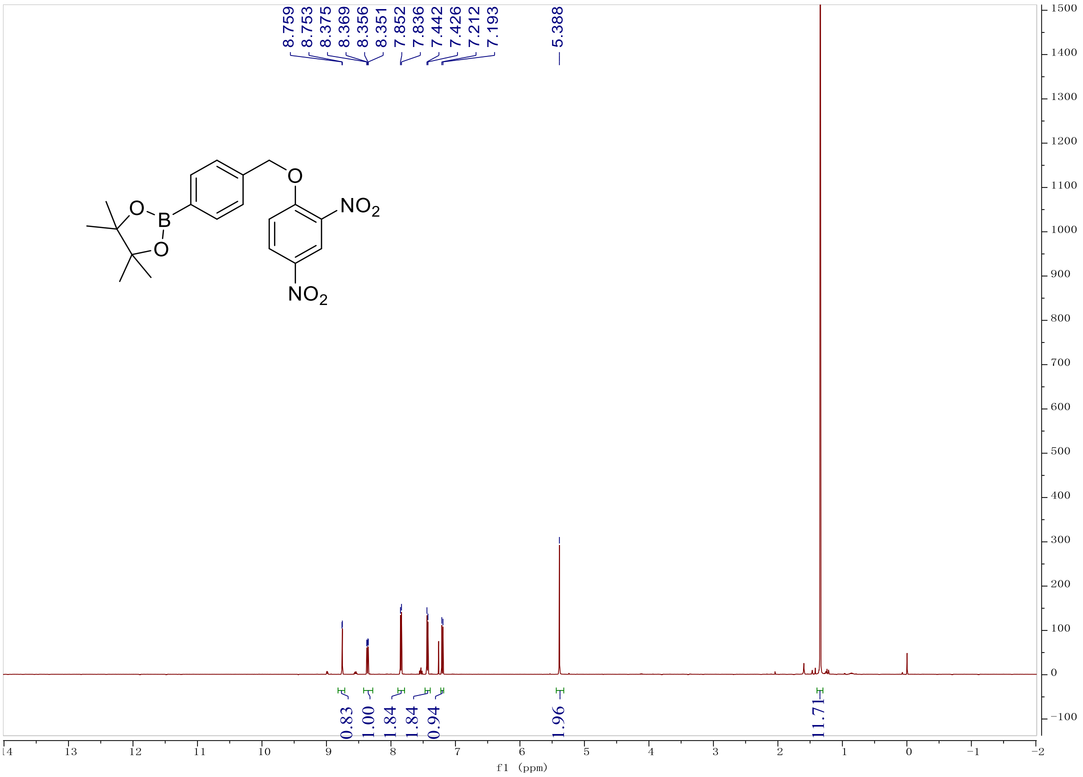

13: ^{13}C-NMR (151MHz, $CDCl_3$)

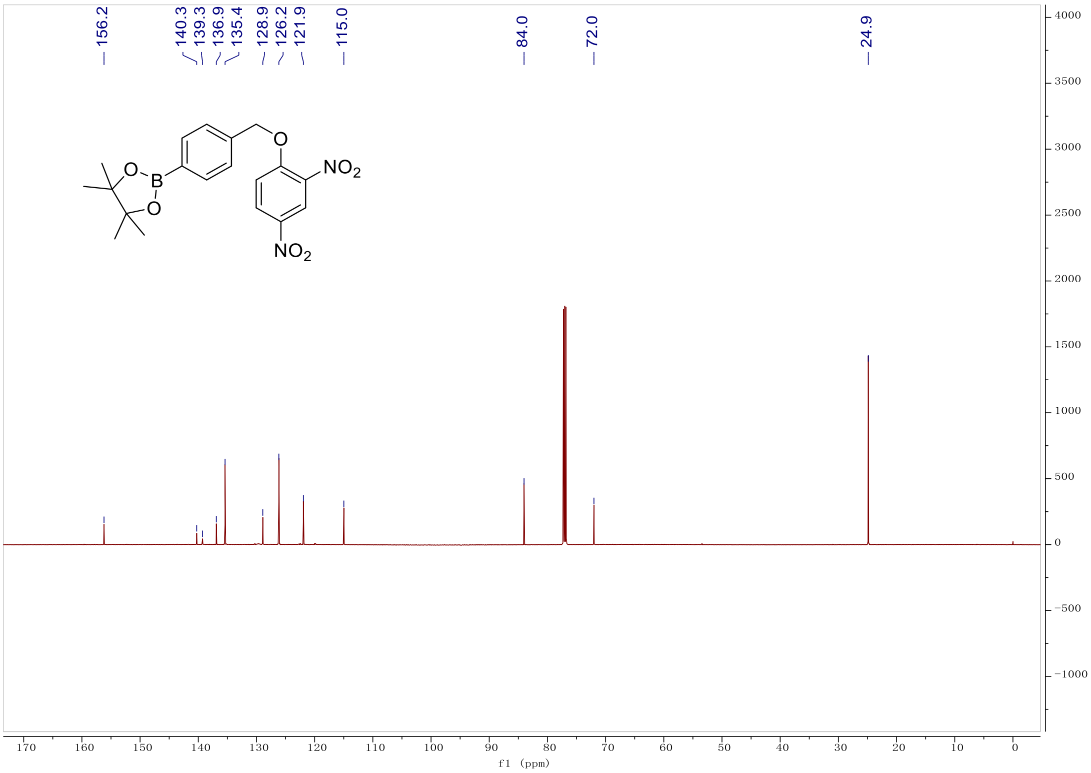

13: DEPT135 (151 MHz, $CDCl_3$)

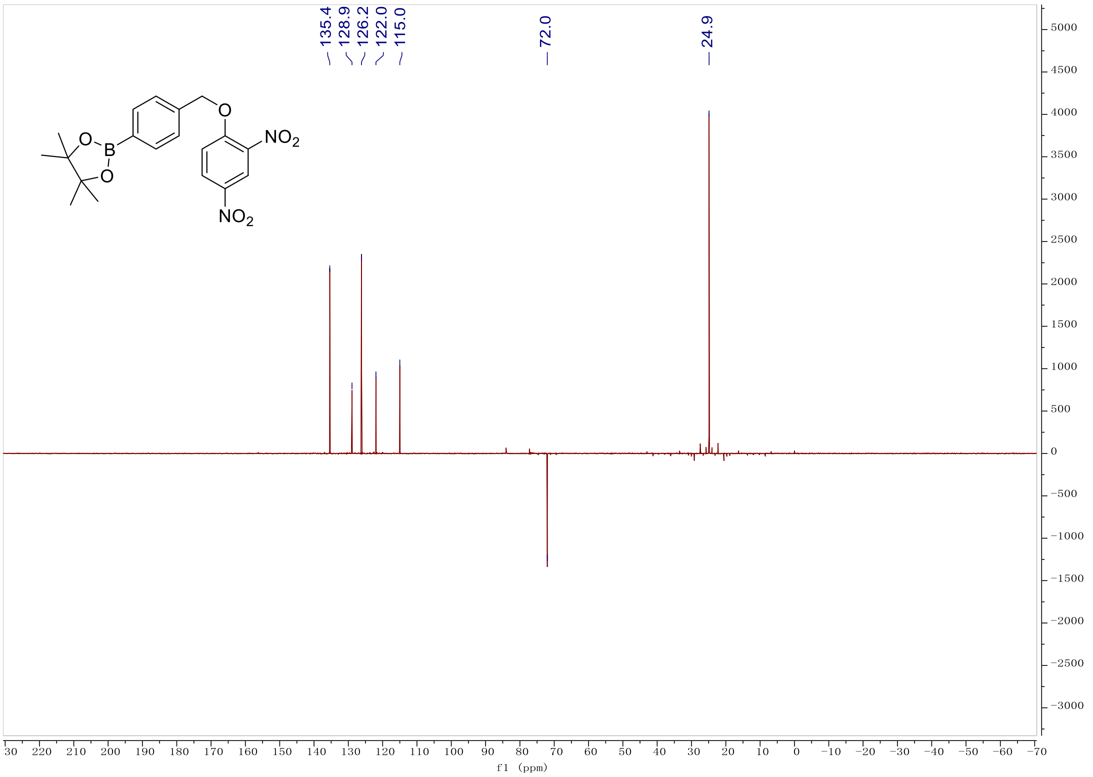

6. References

1. Keppler, A.; Kindermann, M.; Gendreizig, S.; Pick, H.; Vogel, H.; Johnsson, K., Labeling of fusion proteins of O6-alkylguanine-DNA alkyltransferase with small molecules in vivo and in vitro. *Methods* **2004,** *32* (4), 437-44.

2. Keppler, A.; Gendreizig, S.; Gronemeyer, T.; Pick, H.; Vogel, H.; Johnsson, K., A general method for the covalent labeling of fusion proteins with small molecules in vivo. *Nat. Biotechnol.* **2003,** *21* (1), 86-9.

3. Wang, H.; Li, W. G.; Zeng, K.; Wu, Y. J.; Zhang, Y.; Xu, T. L.; Chen, Y., Photocatalysis Enables Visible-Light Uncaging of Bioactive Molecules in Live Cells. *Angew. Chem. Int. Ed.* **2019,** *58* (2), 561-565.

4. Li, M.; Xia, J.; Tian, R.; Wang, J.; Fan, J.; Du, J.; Long, S.; Song, X.; Foley, J. W.; Peng, X., Near-Infrared Light-Initiated Molecular Superoxide Radical Generator: Rejuvenating Photodynamic Therapy against Hypoxic Tumors. *J. Am. Chem. Soc.* **2018,** *140* (44), 14851-14859.

5. Gandin, E.; Lion, Y.; Van de Vorst, A., Quantum Yield of Singlet Oxygen Production by Xanthene Derivatives. *Photochem. Photobio.* **1983,** *37* (3), 271-278.

6. Lancaster, J. R., Diffusion of free nitric oxide. *Methods Enzymol.* **1996,** *268*, 31-50.

7. Winterbourn, C. C., Reconciling the chemistry and biology of reactive oxygen species. *Nat. Chem. Biol.* **2008,** *4* (5), 278-86.

8. Wang, Y.; Branicky, R.; Noë, A.; Hekimi, S., Superoxide dismutases: Dual roles in controlling ROS damage and regulating ROS signaling. *J. Cell Biol.* **2018,** *217* (6), 1915-1928.

9. Chen, H.; Chen, T.-Y., Probing Oxidant Effects on Superoxide Dismutase 1 Oligomeric States in

Live Cells Using Single-Molecule Fluorescence Anisotropy. *Chem. Biomed. Imaging* **2023,** *1* (1), 49-57.
10. Wang, P.; Tang, W.; Li, Z.; Zou, Z.; Zhou, Y.; Li, R.; Xiong, T.; Wang, J.; Zou, P., Mapping spatial transcriptome with light-activated proximity-dependent RNA labeling. *Nat. Chem. Biol.* **2019,** *15* (11), 1110-1119.
11. Rembiałkowska, N.; Dubińska-Magiera, M.; Sikora, A.; Szlasa, W.; Szewczyk, A.; Czapor-Irzabek, H.; Daczewska, M.; Saczko, J.; Kulbacka, J., Doxorubicin Assisted by Microsecond Electroporation Promotes Irreparable Morphological Alternations in Sensitive and Resistant Human Breast Adenocarcinoma Cells. *Appl. Sci.* **2020,** *10* (8).
12. Ye, M. Z.; Han, Y. X.; Tang, J. B.; Piao, Y.; Liu, X. R.; Zhou, Z. X.; Gao, J. Q.; Rao, J. H.; Shen, Y. Q., A Tumor-Specific Cascade Amplification Drug Release Nanoparticle for Overcoming Multidrug Resistance in Cancers. *Adv. Mater.* **2017,** *29* (38).
